# Evolution and Taxonomic Significance of Seed Micromorphology in *Impatiens* (Balsaminaceae)

**DOI:** 10.3389/fpls.2022.835943

**Published:** 2022-02-16

**Authors:** Yong-Xiu Song, Shuai Peng, Fredrick Munyao Mutie, Hui Jiang, Jing Ren, Yi-Yan Cong, Guang-Wan Hu

**Affiliations:** ^1^College of Life Sciences, Hunan Normal University, Changsha, China; ^2^Core Botanical Gardens/Wuhan Botanical Garden, Chinese Academy of Sciences, Wuhan, China; ^3^Sino-Africa Joint Research Center, Chinese Academy of Sciences, Wuhan, China; ^4^University of Chinese Academy of Sciences, Beijing, China

**Keywords:** character evolution, *Impatiens*, seed micromorphology, SEM, taxonomy

## Abstract

*Impatiens* is one of the most species-rich genera of angiosperms, with more than 1,000 species distributed in Eurasia and Africa. Previous studies have shown that seeds of *Impatiens* display enormous morphological diversity, and seed micromorphology may contribute to the classification of those species. However, the micromorphological seed coat characteristics of *Impatiens* seeds have not been systematically studied. In this study, we examined 117 *Impatiens* seeds from two subgenera and seven sections, and analyzed the seed characters of shape, primary ornamentation, secondary ornamentation, anticlinal cell wall, and periclinal cell wall. We discovered that, according to the different shapes of ornamentation, seed coat can be divided into three types, viz, reticulate type, protrusive type, appendicular type, and that they can be further subdivided into 10 subtypes. In addition, the characteristics of seed coat ornamentation with taxonomic significance in this genus are discussed. We reconstructed the ancestral states of the above seed characters of the *Impatiens* using the maximum likelihood approach based on the reconstructed phylogenetic framework. The seed character reconstruction showed that the seed shape, primary ornamentation, and anticlinal cell wall could be identified as unambiguous, while other characters were ambiguous in the last common ancestor of *Impatiens*. Reconstruction of important seed morphological characters showed that the secondary ornamentation possesses high plasticity, and the primary ornamentation has high homology. In addition, we inferred the evolutionary trends of seed ornamentation and found that the seed coat ornamentation of *Impatiens* experienced a complex evolutionary process from a reticulate type into more complex types. We also discussed the relationship between seed coat diversity vs. environmental adaptation and seed dispersal in *Impatiens*.

## Introduction

Balsaminaceae comprises two genera, the *Hydrocera* Blume ex Wight and Arnott (Blume, 1834, p. 140) and *Impatiens* Linnaeus (1753, p. 937). In contrast to the monotypic *Hydrocera* Blume ex Wight and Arnott (Fischer, 2004; Chen Y. L. et al., 2007), *Impatiens* L. is one of the largest genera of angiosperms, with more than 1,000 species, mainly distributed in the tropical and subtropical regions. Five hotspots of *Impatiens* diversity are recognized: tropical Africa (c. 131 spp.) (Abrahamczyk et al., 2016; Janssens et al., 2018), Madagascar (c. 260 spp.), south India and Sri Lanka (c. 200 spp.), Sino-Himalaya (c. 140 spp.), and Southeast Asia (c. 300 spp.) (Grey-Wilson, 1980; Yu, 2012; Abrahamczyk and Fischer, 2015; Ruchisansakun et al., 2016; Fischer et al., 2017). *Impatiens* species are distributed from sea level to about 4,000 m a.s.l. and occur in diverse habitats such as in forest understories, roadside ditches, valleys, abandoned fields, along streams and in seepages, favoring mesic or wet conditions, with few exceptions (Yu et al., 2015).

Most *Impatiens* species are annual or perennial herbs, but a few are shrubs or subshrubs. The stem is usually fleshy and succulent, making collected specimens difficult to dry and preserve (Chen Y. L. et al., 2007). The genus is distinguished by zygomorphic flowers with tremendous diversity in flower color and morphology, with their sepals being funnelform or saccate, where the navicular lower sepal may have or may lack a spur, and their lateral petals are always united into pairs to form lateral united petals. The fruit is a fleshy, explosive capsule, which dehisces elastically along the valves when ripe to disperse the seeds (Chen, 2007; Yu et al., 2015). Due to their beautiful flowers and the long flowering period, several species of *Impatiens* are popular ornamental plants, with *I. walleriana* Hook. f., *I. hawkeri* W. Bull, and *I. balsamina* L. being the most popular ornamental species, while *I. walleriana* Hook. f. has been commercially grown in Serbia for many years (Duric et al., 2021).

However, *Impatiens* L. is notoriously difficult to classify morphologically (Hooker, 1908; Grey-Wilson, 1980), and the semi-succulent stems, fleshy leaves, and extremely fragile flowers make it challenging to prepare good herbarium specimens (Tan et al., 2015). Warburg and Reiche (1895) provided a complete sub-classification system of the *Impatiens* and divided the genus into two subgenera, *Acaulimpatiens* and *Caulimaptiens*, based on the placement of leaves. Thereafter, regional studies on the classification of *Impatiens* have been conducted. For example, Grey-Wilson (1980) conducted a systematic taxonomic study of 110 species of *Impatiens* from Africa. A comprehensive taxonomic study of the *Impatiens* in China was conducted by Chen (2001), which is included in the flora of China. However, with the discovery of new species in the recent years, it has become difficult to classify *Impatiens* based on traditional classification systems. With the development of molecular biology, molecular phylogenetics can help to better solve the taxonomic challenges and genetic relationship of *Impatiens*. Molecular systematics and phylogeny studies of *Impatiens* have been reported in recent years (Fujihashi et al., 2002; Yuan et al., 2004; Janssens et al., 2006, 2007, 2012). To understand the phylogenetic relationship of *Impatiens*, a phylogenetic tree that was comprehensively sampled from all over the world was constructed by Yu et al. (2015), where based on both morphological and molecular evidence, the genus was divided into two subgenera, *I.* subgen. *Clavicarpa* and *I.* subgen. *Impatiens*, which are widely recognized. *I.* subgen. *Impatiens* was further subdivided into seven sections: *Semeiocardium, Racemosae, Fasciculatae, Impatiens, Tuberosae, Scorpioidae*, and *Uniflorae* (Yu et al., 2015).

At present, we mainly rely on flower morphology to identify *Impatiens* species. However, its flowers are very fragile, and most of the distinguishing features, such as color and shape, gradually disappear in herbarium specimens. Compared with flowers, the surface characteristics of seeds are less affected by the environment and are thus more stable and conserved (Chen, 2007); since from fertilization to maturation of fruits, the seed develops in a relatively independent enclosure. The importance of seed micromorphology for classification has long been recognized (Hooker and Thomson, 1860), which can provide valuable information and reliable evidence for the classification of species and a better understanding of interspecific relationships (Barthlott, 1984; Chen W. et al., 2007; Cai et al., 2013). The scanning electron microscope (SEM) enables observation and study of small-sized structures that were previously impossible to examine using conventional light microscopy (Heywood, 1969; Stace, 1992). For this reason, it is generally used for the examination of surface morphology and the size of biology. The progress in microscopic imaging using SEM constitute additional approaches to determine the boundaries between organisms (Brisson and Peterson, 1976; Godfray and Knapp, 2004). There are some fundamental studies on seed testa characteristics in genus *Impatiens*. For example, Song et al. (2005) examined the seed micromorphology of 38 species of *Impatiens* mostly from Southwest China and divided them into four morphological types, viz, laevigate, granulate, reticulate, and protrusive. Utami and Shimizu (2005) examined the seed micromorphology of 65 species of *Impatiens* and discussed systematic problems. Cai et al. (2013) examined the seeds of 24 species of *Impatiens* from China and divided them into two major types: reticulate type and protrusive type, and briefly discussed the systematic taxonomic significance of the micromorphology of the seed coat. Furthermore, fragmented studies on the seed coat of *Impatiens* were investigated by Lu and Chen (1991), Tian (2004), Chen (2007), Fischer et al. (2017), Yin (2018), Najberek et al. (2020), and Rewicz et al. (2020a,b). However, due to insufficient sampling, it was hard for these studies to provide a comprehensive conclusion on the overall variation in seed coat micromorphology in *Impatiens*. Moreover, these studies described only the main types of seed coat in *Impatiens*, without conducting an in-depth investigation. In addition, there is scanty information about seed character evolution in *Impatiens*.

In the present study, we systematically examined seed morphology and testa sculpture of 117 species of *Impatiens* from two subgenera and seven sections, covering all representative eight groups. The aims of our study are to (1) increase taxon sampling in the *Impatiens* to evaluate the taxonomic and systematic significance of seed morphological characters in a phylogenetic context (2) divide the morphological types of testa ornamentation and discuss the potential taxonomic significance in *Impatiens*, (3) explore character evolution of seed traits of *Impatiens*, detect most recent common ancestor and synapomorphy, and infer the evolutionary trends of seed ornamentation.

## Materials and Methods

### Species Sampling, Sequencing, and Phylogenetic Analyses

A total of 117 species of *Impatiens* were used for phylogenetic analysis, covering most of the geographical range of *Impatiens* and including all representatives of the eight infrageneric groups. DNA sequence data of 81 species were downloaded from GenBank, while the other sequences of 36 species were newly sequenced (NCBI GenBank numbers are given in Supplementary Table 1). *Hydrocera triflora* was selected as outgroup.

Nuclear ribosomal ITS (Yuan et al., 2004) and plastid *atpB-rbcL* (Janssens et al., 2006) were used to reconstruct the phylogenetic relationships. Genomic DNA was extracted from silica-gel dried leaf material or from herbarium specimens using Mag-MK Plant Genomic DNA extraction kits (Sangon Biotech, Shanghai). PCR product sequencing was conducted by TSINGKE Biological Technology.

Phylogenetic analysis was carried out in PhyloSuite v. 1.2.2 (Zhang et al., 2020). The sequence was assembled and edited using MEGA v. 7.0.26 (Kumar et al., 2018). Sequences were aligned using MAFFT v. 7.222 (Katoh and Standley, 2013). The best-fit DNA substitution models were selected with ModelFinder (Kalyaanamoorthy et al., 2017). Bayesian inference (BI) was performed using MrBayes v. 3.2.7a (Ronquist et al., 2012) under the GTR + I + G (ITS) and GTR + G (*atpB-rbcL*) model (2 parallel runs, 10 million generations, and sampled every 1,000 generations), in which the initial 25% of sampled data were discarded as burn-in. Maximum likelihood (ML) phylogenies were inferred using IQ-TREE (Nguyen et al., 2015) for 1,000 ultrafast bootstraps (Minh et al., 2013) under the GTR + I + G (ITS) and GTR + G (*atpB-rbcL*) model as well as the Shimodaira-Hasegawa-like approximate likelihood-ratio test (Guindon et al., 2010).

### Micromorphology of Seed

**FIGURE 1:**
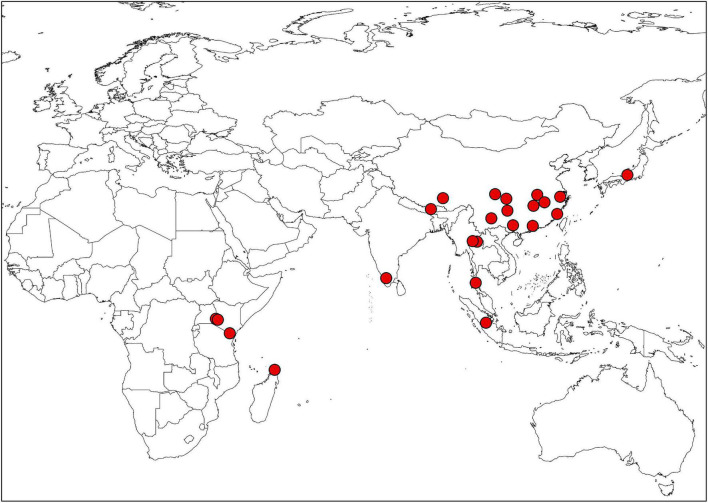
The distribution map of *Impatiens* in this study.

Extensive field investigations were conducted to collect specimens and seed materials of *Impatiens* (voucher specimens are shown in Supplementary Table 2; the distribution map for all species is provided in Figure 1). Mature capsules that were ready to explode at the touch were selected to observe the morphological characteristics of seed epidermis, and the seeds were collected from the field together with the specimens. Eight to ten plump seeds of each *Impatiens* from different individuals were carefully selected for dehydration; these mature seeds were dried and dehydrated with different concentrations of alcohol (60% for 3 h, 70% for 3 h, 80% for 3 h, 90% for 3 h, 95% for 3 h, and 100% overnight). Then, randomly selected six dried seeds were mounted on stubs with double-sided adhesive tape and sputter coated with gold. Coated seeds were then examined and photographed using the JSM-IT500 SEM. The coated samples were imaged at 30 × –50 × magnifications for the whole view, 200 × and 800 × magnifications for the same lateral partial view of seeds at an accelerating voltage of 20 kV and a working distance of 10 mm. Morphological characters of the seeds were described following Lu and Chen (1991), Liu et al. (2004), and Song et al. (2005).

### Seed Character Evolution

We conducted ancestral state reconstruction for five seed characters that were considered significant for the taxonomy of *Impatiens*. These characters were mapped and coded as follows: (1) seed shape: 0, ellipsoid; 1, oblong; 2, ovate; 3, subspheroid; (2) primary ornamentation: 0, reticulate type; 1, protrusive type; 2, appendicular type; (3) secondary ornamentation: 0, fine reticulate; 1, areolate; 2, striate; 3, digitiform; 4, clustered; 5, carinate; 6, squamalate; 7, threaded; 8, cristate; 9, granulate; (4) periclinal cell wall: 0, concave; 1, convex; (5) anticlinal cell wall: 0, irregularly curved, 1, straight; 2, unclear. The matrix (Supplementary Table 3) was compiled for seed characters of the 117 species in *Impatiens* from SEM photographs, specimens, and the previous literature studies (Supplementary Table 4). Estimation of ancestral states was conducted under maximum likelihood (ML) optimization of character evolution. We traced and visualized the character mapping using Mesquite v. 3.51 (Maddison and Maddison, 2018) using the Markov k-state 1 parameter model (Mk1). All the characters were treated as unordered and equally weighted. The analysis was done using the “Trace Character History” option and the ML approach.

## Results

### Phylogenetic Analyses

**FIGURE 2:**
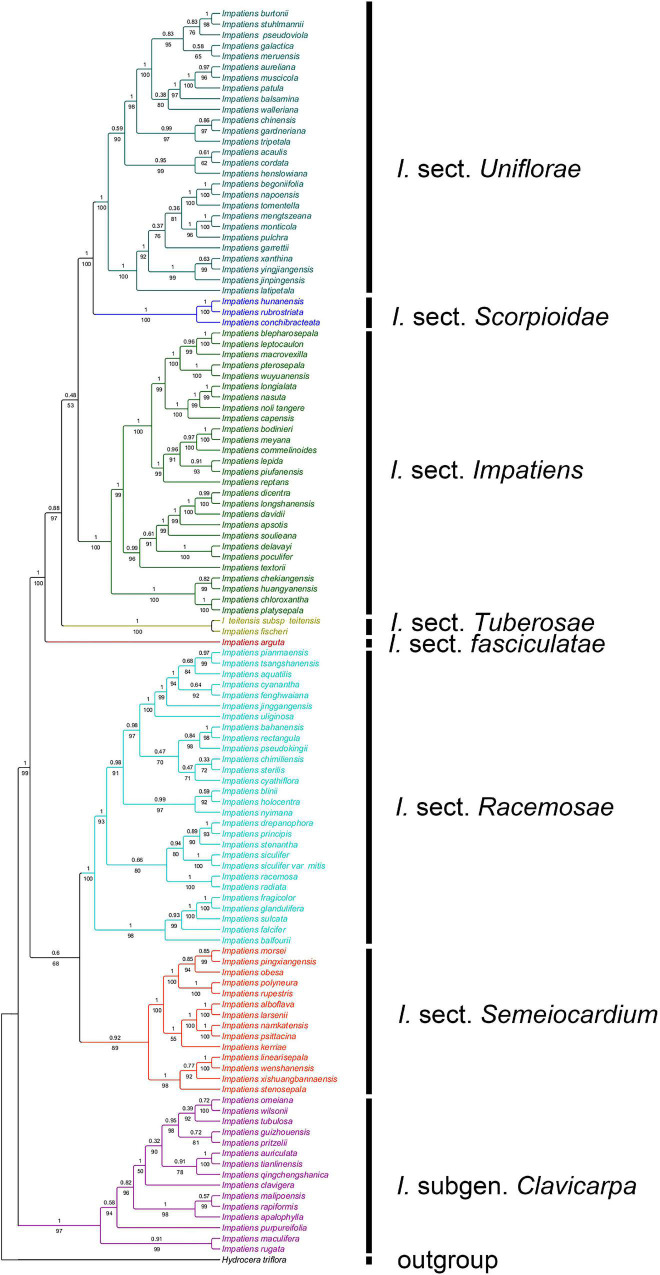
The phylogenetic tree inferred from maximum likelihood (ML) and Bayesian based on a combined dataset of ITS and *atpB-rbcL* sequences. Values below the branches are maximum likelihood bootstrap supports, and above the branches are Bayesian posterior probabilities.

A total of 189 sequences were used for *Impatiens* in this study. The final aligned positions of the combined ITS and *atpB-rbcL* data set were 1,657 base pairs (bps): ITS with 758 bps and *atpB-rbcL* with 899 bps. The reconstructed phylogenetic tree is largely similar to the tree recovered by Yu et al. (2015). It contains *I.* subgen. *Clavicarpa* and subgen. *Impatiens*, and the latter can be divided into several sections (Figure 2).

### Micromorphology of Seed

**FIGURE 3:**
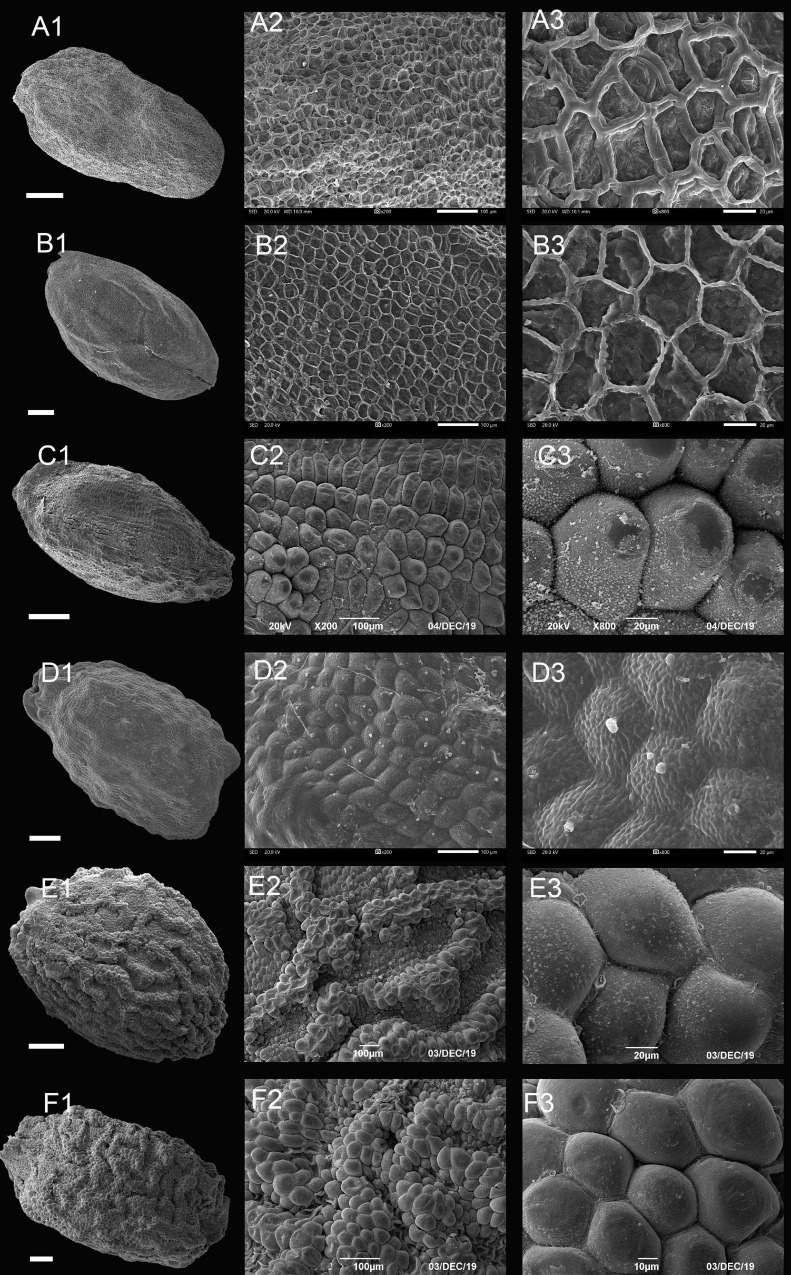
Scanning electron microscope images of seeds from reticulate type of *Impatiens*. **(A1–F1)** Whole view; **(A2–F2,C3–F3)** Partial view. **(A1–A3)** *Impatiens rapiformis*, **(B1–B3)** *Impatiens maculifera*, **(C1–C3)** *Impatiens cyanantha*, **(D1–D3)** *Impatiens sulcata*, **(E1–E3)** *Impatiens pterosepala*, **(F1–F3)** *Impatiens leptocaulon*. Scale bars: **(A1–E1)** = 500 μm, **(F1)** = 200 μm.

**FIGURE 4:**
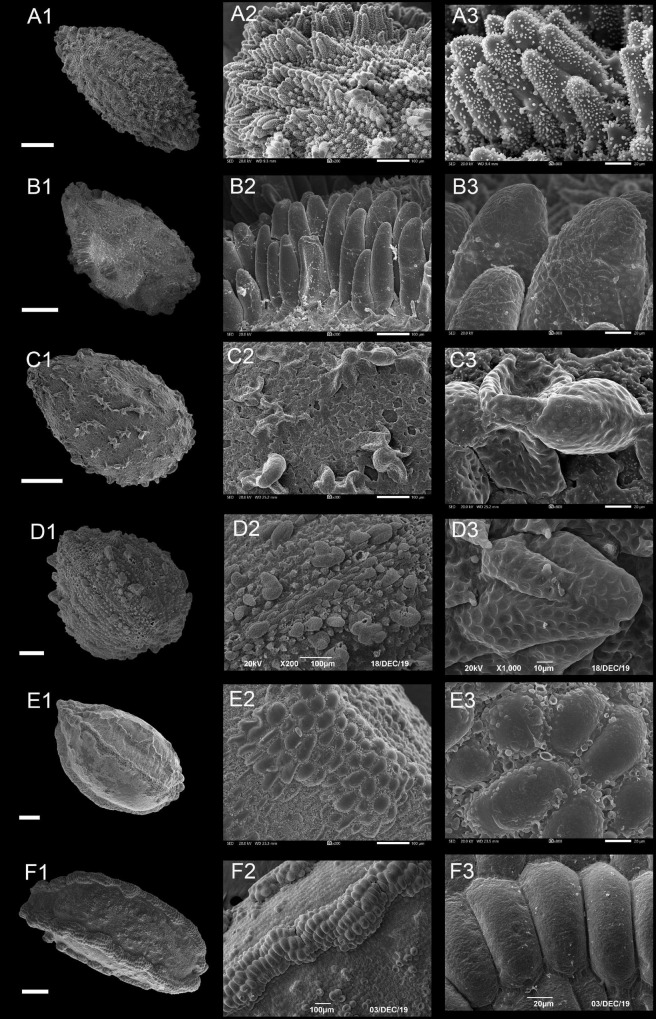
Scanning electron microscope images of seeds from protrusive type of *Impatiens*. **(A1–F1)** Whole view; **(A2–F2,C3–F3)** Partial view. **(A1–A3)** *Impatiens huangyanensis*, **(B1–B3)** *Impatiens cyathiflora*, **(C1–C3)** *Impatiens principis*, **(D1–D3)** *Impatiens drepanophora*, **(E1–E3)** *Impatiens longialata*, **(F1–F3)** *Impatiens noli-tangere*. Scale bars: **(A1–C1,E1,F1)** = 500 μm, **(D1)** = 200 μm.

**FIGURE 5:**
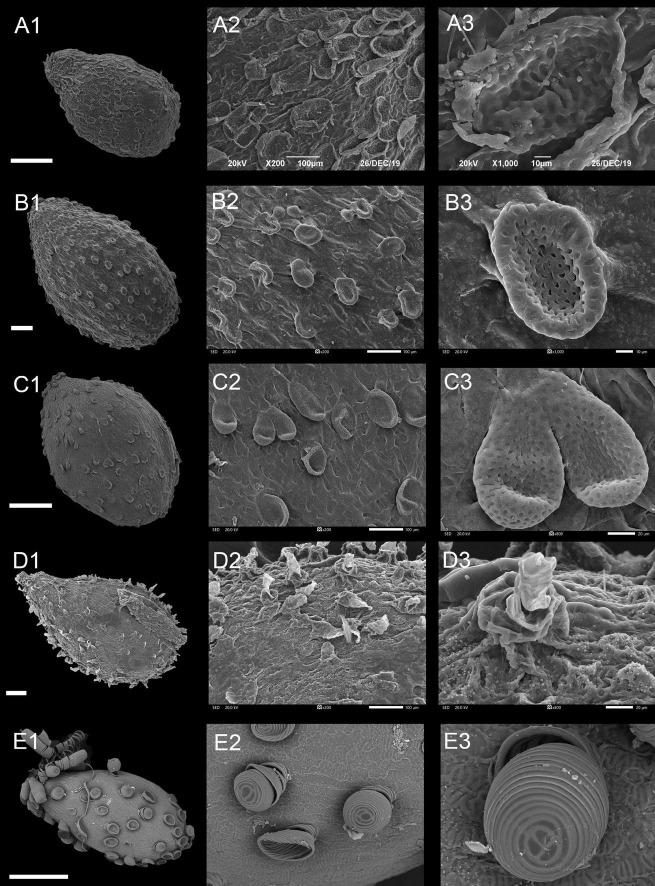
Scanning electron microscope images of seeds from appendicular type of *Impatiens*. **(A1–E1)** Whole view; **(A2–E2,C3–E3)** Partial view. **(A1–A3)** *Impatiens monticola*, **(B1–B3)** *Impatiens jinpingensis*, **(C1–C3)** *Impatiens latipetala*, **(D1–D3)** *Impatiens psedoviola*, **(E1–E3)** *Impatiens xanthina*. Scale bars: **(A1,C1,E1)** = 500 μm, **(B1,D1)** = 200 μm.

**FIGURE 6:**
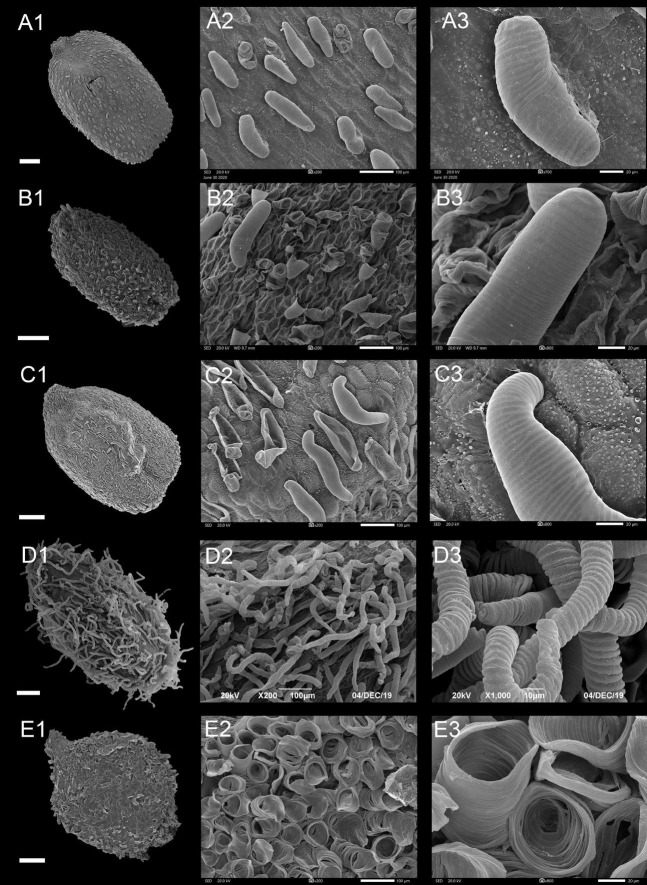
Scanning electron microscope images of seeds from appendicular type of *Impatiens*. **(A1–E1)** Whole view; **(A2–E2,C3–E3)** Partial view. **(A1–A3)** *Impatiens rupestris*, **(B1–B3)** *Impatiens obesa*, **(C1–C3)** *Impatiens polyneura*, **(D1–D3)** *Impatiens tripetala*, **(E1–E3)** *Impatiens burtonii*. Scale bars: **(A1–C1,E1)** = 500 μm, **(D1)** = 200 μm.

We obtained seed morphological data for a total of 117 *Impatiens* species, 70 of which are reported for the first time (Figures 3–6 and Supplementary Figures 1–11), and the rest from published data listing in Supplementary Table 4. Morphological characters of the seeds are summarized in Supplementary Table 5. We found that the seeds of *Impatiens* were highly variable in size and shape, especially the seed coat ornamentation, while there were little changes in the periclinal cell wall and the anticlinal cell wall. Seed dimensions ranged from 0.89 mm × 0.62 mm (length × width) (*I. acaulis*) to 7.77 mm × 2.93 mm (*I. auriculata*); the outlines were oblong, ellipsoid, subspheroid, or ovate; the periclinal cell wall was concave or convex, and the anticlinal cell wall was irregularly curved, straight, or unclear. Based on the complex ornamentation, the epidermal cells of the seed coat can be divided into three principal morphological types: namely, reticulate type, protrusive type, and appendicular type. Each morphological type can be further divided into the following subtypes.

#### Type I: Reticulate Type

The outer periclinal wall of the epidermal cells of the seed coat was concave or slightly convex, forming a conspicuous reticulate ornamentation, and the meshes had irregular shape that varied in size (Figure 3 and Supplementary Figures 1–7A). Based on the raised or sunken mesh, three subtypes can be distinguished: fine reticulate subtype, areolate subtype, and striate subtype.

##### Type IA: Fine Reticulate Subtype

The outer periclinal wall of epidermal cells of the seed coat is mostly irregular polygons, rete ridges are obvious, the periclinal cell walls are concave, and anticlinal cell walls are convex (Figures 3A,B and Supplementary Figures 1–3D). All species of *I.* subgen. *Clavicarpa* belong to this group (Figures 3A,B). The seeds of this subgenus are all ellipsoid in shape, and all are large, except for the *I. omeiana*. However, these seeds vary in size of the mesh, the internal structure of the mesh, and the width of the ridge, which varies from 3.02 to 9.65 μm. This subtype is also distributed in other sections of *Impatiens* (sect. *Semeiocardium*, sect. *Impatiens*).

##### Type IB: Areolate Subtype

The outer periclinal walls of epidermal cells of the seed coat are alveolar and bulged to form reticulation, evenly distributed across the entire seed coat, and the rete ridges are sunken (Figures 3C,D and Supplementary Figures 3E,F, 4A). This subtype is distributed in *I.* sect. *Racemosae* and sect. *Uniflorae*. However, the seed’s shape and size of this subtype show significant diversity, with the shape of the seeds ranging from ellipsoid, subspheroid, to ovate, while the size varies from 2.1 mm × 1.5 mm to 3.72 mm × 2.48 mm. At the same time, the morphology of convex periclinal cell walls varies among species (Figures 3C,D).

##### Type IC: Striate Subtype

This subtype is similar to subtype IB, except that the seed coat epidermal cells are elevated slightly higher and form irregular bend stripes (Figures 3E,F and Supplementary Figures 4–6, 7A). This subtype is mostly found in *I.* sect. *Impatiens* (Figures 3E,F). There are also a few distributions in sect. *Semeiocardium*, sect. *Racemosae*, and sect. *Scorpioidae*. The sizes of the seeds in this subtype range from 1.7 mm × 1.1 mm to 4.3 mm × 2.9 mm and are oblong, ellipsoid to ovate in shape.

#### Type II: Protrusive Type

In this type, a few or most epidermal cells of the seed coat had obvious protrusions and formed projections of varying shapes (Figure 4 and Supplementary Figures 7B–10C). Based on the shape of the protrusions, the following three subtypes can be provided.

##### Type IIA: Digitiform Subtype

Most of the protruding epidermal cells of the seed coat appeared finger like, and the surface of protrusions had granular appendages or holes (Figures 4A,B and Supplementary Figures 7B–10B). This subtype appears in *I.* sect. *Impatiens* and sect. *Racemosae*. The shape of the seeds in sect. *Racemosae* is variable, ranging from oblong, ellipsoid to ovate, with the size ranging from small, medium, to large. However, the shape of the seeds in sect. *Impatiens* is ellipsoid, and medium in size. In this subtype, the protrusions have numerous cuticular granules on their lateral surface in some species such as *I. huangyanensis* (Figure 4A), *I. chekiangensis* (Supplementary Figure 9E), whereas, in the other species of this subtype, the protrusions have numerous pits (Figure 4B).

##### Type IIB: Clustered Subtype

Most of the epidermal cells protrude from the surface of the seed coat, and several mound-shaped protrusions cluster together (Figures 4C,D). This subtype was found in *I. drepanophora* and *I. principis*, which belong to *I.* sect. *Racemosae*. Seeds in this subtype are ovate in shape. In *I. drepanophora* and *I. principis*, the protrusions have numerous pits (Figures 4C,D).

##### Type II C: Carinate Subtype

In this subtype, 4–5 rows seed coat epidermal cells are elevated significantly higher than the others and are arranged in ridges, forming keel-like protrusions (Figures 4E,F and Supplementary Figure 10C). This subtype appears in *I. longialata* (Figure 4E), *I. noli-tangere* (Figure 4F), *I. nasuta*, and *I. capensis*, which belong to the *I.* sect. *Impatiens*. The seeds are ellipsoid and large. The periclinal cell walls are granulated in *I. longialata*, while the periclinal cell walls are smooth in *I. noli-tangere* (Figure 4F3).

#### Type III: Appendicular Type

Some of the epidermal cells of the seed coat have different morphological appendages (Figures 5, 6 and Supplementary Figures 10E–11). According to the shapes of the appendages, the following four subtypes can be divided.

##### Type IIIA: Squamalate Subtype

A few epidermal cells of the seed coat displayed squamalate protrusions, and scale-like protrusions with small holes sparsely covering the entire seed coat (Figures 5A–C and Supplementary Figures 10E–11A). This subtype is specific to *I.* sect. *Uniflorae*. The seeds of this subtype vary from 1.40 mm × 0.96 mm to 3.07 mm × 2.19 mm in size, and are ovate or ellipsoid in shape, except for the *I. napoensis* and *I. garrettii*, whose pits are conspicuous on the squamalate protrusions.

##### Type IIIB: Cristate Subtype

Compared with the squamalate subtype, a few epidermal cells of the seedcoat of this subtype are crest like, and are evenly or unevenly distributed on the seed coat (Figures 5D,E and Supplementary Figures 11B,E,F). This subtype is distributed in some species of *I.* sect. *Uniflorae* and sect. *Semeiocardium*. The seeds are all ovate, and the seed size is medium or small. Fine cuticular granules densely cover the non-protruding epidermal cells in *I. walleriana* and *I. aureliana* (Supplementary Figure 11). The protrusions of *I. xanthina* are spirally thickened, but no cuticular granules were seen.

##### Type IIIC: Threaded Subtype

Protrusions of the epidermal cells of the seed coat are threaded, and are tubular or filamentous, sparsely or evenly distributed on the entire seed surface (Figure 6 and Supplementary Figures 11C,D). This subtype occurs in *I.* sect. *Semeiocardium* and sect. *Uniflorae*. The shape of the seeds in this subtype ranges from subspheroid, ellipsoid, oblong to ovate, while the size ranges from small, medium, to large. In sect. *Semeiocardium*, the surface of some epidermal cells has tubular threaded-like appendages, where these appendages in *I. obesa* are 213.47-μm long (Figure 6B), and 246.77 μm in *I. polyneura* (Figure 6C). Unlike the vast majority of *Impatiens* seed coat, the surface of the seed coat in the *I. tripetala* is densely covered with filamentous thread-like appendages (Figure 6D).

##### Type IIID: Granulate Subtype

In this subtype, the surface of the seed coat was almost evenly covered with granulate protrusions. This subtype was found in *I.* sect. *Semeiocardium* and sect. *Uniflorae*. Seeds are subspheroid or ellipsoid in this subtype. Under high magnification (×800), each granule is a flower bud-like protrusion with radial pleats in *I. balsamina*.

### Seed Character Evolution

The seed morphological characters, seed shape, primary ornamentation, secondary ornamentation, periclinal cell wall, and anticlinal cell wall were mapped on the molecular tree to reconstruct ancestral states and analyze the correspondence of seed morphological to molecular data. The results showed that the ancestral character state of seed shape, primary ornamentation, and anticlinal cell wall were unambiguous, while the ancestral character state of other seed characters was equivocal. The ancestral states of each character on each node of the phylogeny using maximum likelihood with the MK1 model are provided in Supplementary Table 6.

#### Seed Shape

**FIGURE 7:**
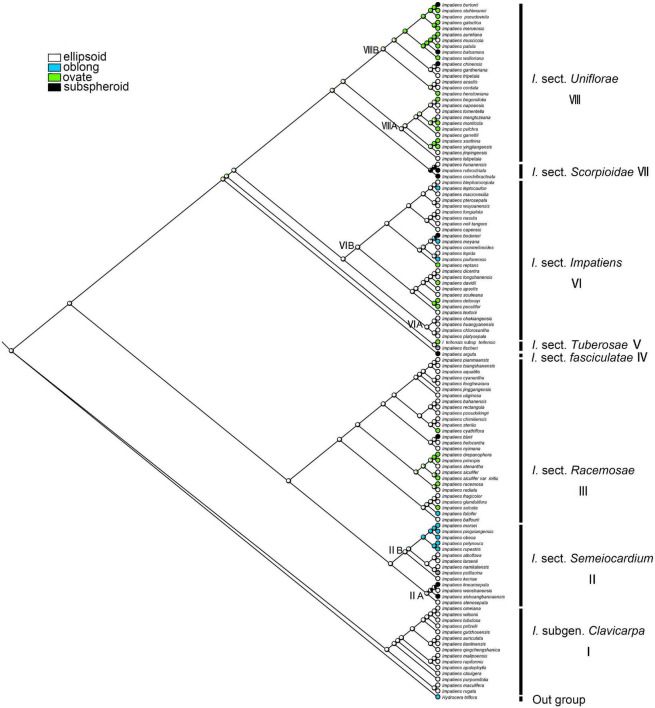
Ancestral reconstruction of the seed shape in *Impatiens* using ML. Four characteristic states of seed shape of *Impatiens* optimized on the BI tree by using different colors.

The ancestral character state of “seed shape” was reconstructed as “ellipsoid” with maximum likelihood (ML = 0.90, Figure 7). These two seed-shape character states (ellipsoid and ovate) alternate in the reconstruction in *I.* sect. *Uniflorae* (Figure 7, clade VIII). Oblong seeds were found to have evolved five times independently, and they appeared in sect. *Semeiocardium*, sect. *Racemosae*, and sect. *Impatiens*; subspheroid seeds were found to have evolved eight times independently, and they appeared in sect. *Uniflorae*, sect. *Racemosae*, sect. *Impatiens*, and sect. *Scorpioidae*.

#### Primary Ornamentation

**FIGURE 8:**
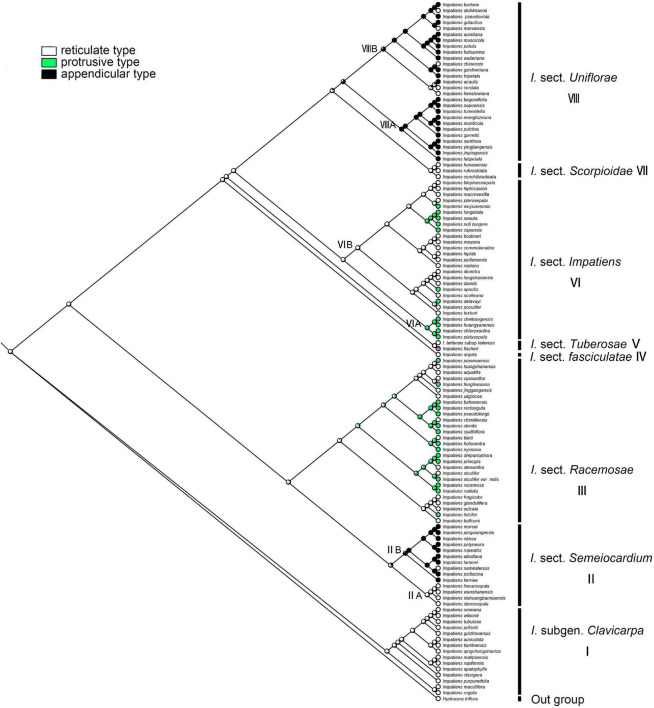
Ancestral reconstruction of the primary ornamentation in *Impatiens* using ML. Three characteristic states of primary ornamentation of *Impatiens* optimized on the BI tree by using different colors.

The ancestral state of primary ornamentation of the common ancestor of the *Impatiens* was found to be the “reticulate type” by maximum likelihood (ML = 0.98, Figure 8); Appendicular type has evolved two times independently in *I.* sect. *Semeiocardium* (Figure 8, clade II) and sect. *Uniflorae*, while the appendicular type is a synapomorphy of the clade VIIIA in sect. *Uniflorae* (ML = 0.96, clade VIII). The derived state of “protrusive type” first occurred in sect. *Racemosae* (Figure 8, clade III) and experienced changes 11 times. Examples of a reversal to the root ancestral state of “reticulate type” were found in *I. cordata*, *I. henslowiana*, *I. chinensis*, *I. muscicola*, and *I. meruensis* (sect. *Uniflorae*) from the derived states of “appendicular type.”

#### Secondary Ornamentation

**FIGURE 9:**
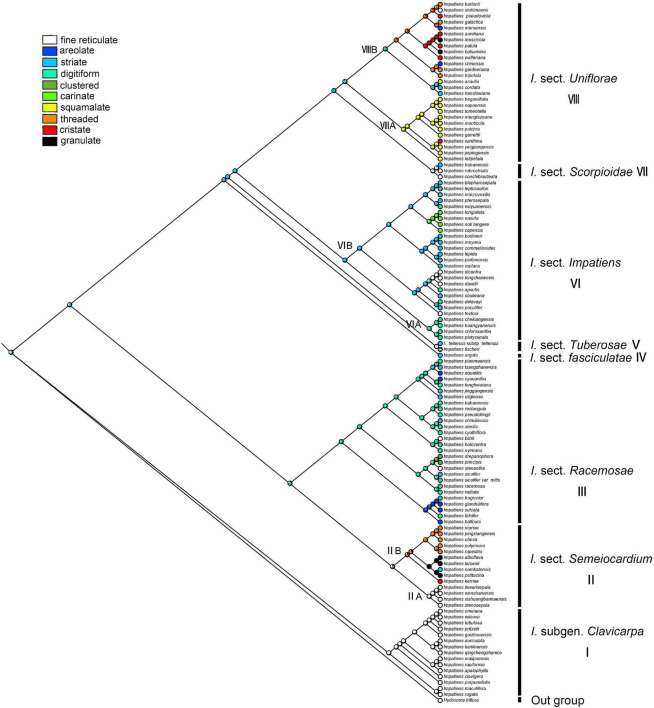
Ancestral reconstruction of the secondary ornamentation in *Impatiens* using ML. Ten characteristic states of secondary ornamentation of *Impatiens* optimized on the BI tree by using different colors.

The secondary ornamentation was ambiguous in the last common ancestor of the *Impatiens*. The “fine reticulate” was inferred as a synapomorphy for *I.* subgen. *Clavicarpa* (ML = 0.98, Figure 9); “squamalate” was a synapomorphy for the clade VIIIA in *I.* sect. *Uniflorae*, and “carinate” was present only in sect. *Impatiens*, and this character state was derived one time in *Impatiens*; the “striate” represented a derived state in sect. *Impatiens* (ML = 0.99, Figure 9); “areolate” and “digitiform” alternate in sect. *Racemosae*, a “threaded” state that evolved independently five times in *Impatiens*, first appearing in sect. *Semeiocardium*. Granulate protrusions of sect. *Semeiocardium* and sect. *Uniflorae* are derived characters.

#### Periclinal Cell Wall

**FIGURE 10:**
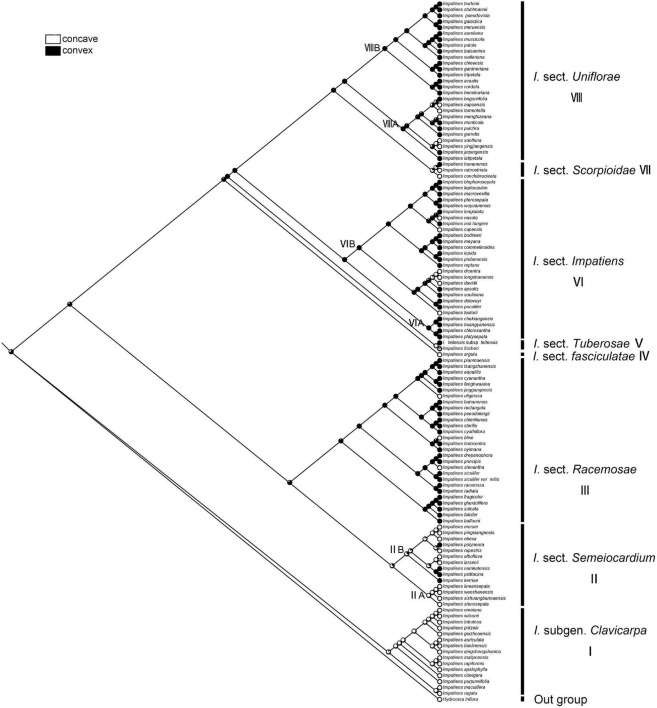
Ancestral reconstruction of the periclinal cell wall in *Impatiens* using ML. Two characteristic states of periclinal cell of *Impatiens* optimized on the BI tree by using different colors.

The character “periclinal cell wall,” which is most commonly convex, can also occur in states of concave. The recovered ancestral state for this character at the root node was equivocal (ML convex = 0.65, concave = 0.35, Figure 10). The “concave” was a synapomorphy for *I.* subgen. *Clavicarpa* (ML = 0.98, Figure 10), and the convex of the periclinal cell wall was inferred as a derived character for *I.* sect. *Racemosae*, sect. *Impatiens*, and sect. *Uniflorae*.

#### Anticlinal Cell Wall

**FIGURE 11:**
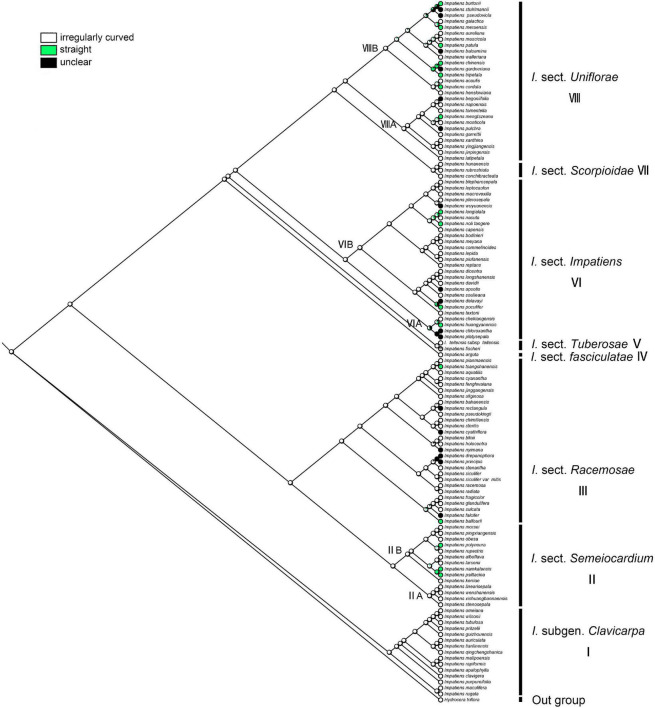
Ancestral reconstruction of the anticlinal cell wall in *Impatiens* using ML. Three characteristic states of anticlinal cell wall of *Impatiens* optimized on the BI tree by using different colors.

The irregular curvature of anticlinal cell wall showed the highest probability as the ancestral state for the last common ancestor of the *Impatiens* (ML = 0.97, Figure 11). Irregularly curved anticlinal cell walls are consistently present in *I.* subgen. *Clavicarpa* (Figure 11, clade I), whereas species of subgen. *Impatiens* (Figure 11, clade VI) have both straight and unclear anticlinal cell walls.

## Discussion

### Taxonomic Significance of Seed Characters

The taxonomic significance of seed micromorphology in *Impatiens* has been examined in previous studies. Song et al. (2005) provided the type of seed coat ornamentation in *Impatiens*, i.e., laevigate, granulate, reticulate, and protrusive, and confirmed that the high diversity of seed coat micromorphology in *Impatiens* is taxonomically informative at species levels. Cai et al. (2013) divided the seed coat ornamentation in *Impatiens* into two types as reticulate type (including fine reticulate, thick reticulate, areolate, carinate, and striate subtypes) and protrusive type (including digitiform, clustered, and squamalate subtypes), and discussed the systematic and phylogenetic implications of seed coat micromorphology in *Impatiens*. The results showed that the diversity of seed coats, such as the shape and depth of lumina, shape of protrusions, and density of derivatives, could be used to identify the species in *Impatiens*.

Here, we systematically investigated the seed micromorphology in *Impatiens* for infrageneric classification with a sample of 117 species. According to the ornamentation of the seed coat, three main types: reticulate type, protrusive type, and appendicular type were distinguished. The threaded subtype in *I. tripetala* (*I.* sect. *Uniflorae*) was observed for the first time. *I. chinensis* was described as having the laevigate seed coat in a previous study (Song et al., 2005), but it was assigned to the reticulate type (the areolate subtype) under high magnification in this study.

The seeds of all species from *I.* subgen. *Clavicarpa* are ellipsoid in shape, belonging to the fine reticulate subtype, coupled with the identification key (perennial, capsule clavate) in previous studies, which is stable and may be used as a criterion for classifying this group. However, the mesh size, mesh ridge width, and the morphology within the mesh show significant variability among species (Figures 3A,B and Supplementary Figure 1), and this character may serve a valid classification of species.

Morphology of seeds in *I.* subgen. *Impatiens* shows higher diversity, compared to subgen. *Clavicarpa*. The shape of the seeds varies from oblong, ellipsoid, sub spheroid to ovate, and the size ranges from 0.89 mm × 0.62 mm to 4.45 mm × 2.54 mm. The seed ornamentation types include reticulate type, protrusive type, appendicular type, and many subtypes. These characters (especially the seed ornamentation) provide important information for intraspecific distinction and delimitation.

In *I.* sect. *Semeiocardium*, the seed coat ornamentation of the clade IIA (Figure 9) is reticulate type (fine reticulate), the seed shape is subspheroid, while the seed coat ornamentation of the clade IIB (Figure 9) is appendicular type (threaded subtype or granulate subtype) except for *I. namkatensis*, and the seed shape is oblong or ellipsoid. Morphologically, the two subclades share the similarity in flower structure to some extent, where both have four lateral sepals. Yu et al. (2015) classified this group into the sect. *Semeiocardium* based on both molecular and morphological evidence, i.e., 4-carpellateovary, obconic capsule (rarely cylindrical). However, only two species of clade IIA were included in this research. In our study, we expanded the sampling of the clade II, and found that the seed coat ornamentation is dramatically different in these two subclades. Therefore, we prefer to divide them into two groups, but the taxonomic treatment for these groups needs further investigation to understand their taxonomic status.

Areolate subtype, clustered subtype, digitiform subtype appeared in *I.* sect. *Racemosae*, and fine reticulate subtype only existed in *I. blini*. In this group, the surface of periclinal cell walls of areolate subtype occurs in two forms: granulated in *I. cyanantha* (Figure 3C) and pleated in *I. sulcate* (Figure 3D); the surface of the protrusions of the clustered subtype has depressions and holes, and the surface of the protrusions of the digitiform subtype shows granular appendages or holes. The seed coat ornamentation showed diversity among *Impatiens* species, which can be used as a tool for classification.

The fine reticulate subtype, striate subtype, digitiform subtype, and carinate subtype were displayed in *I.* sect. *Impatiens*. The fine reticulate subtype appeared in *I. davidii* (Supplementary Figure 2F), *I. longshanensis* (Supplementary Figure 3A), and *I. dicentra* (Supplementary Figure 3B). The distinctive characteristic of these taxa is that the apex of distal lobes of lateral united petals has a filamentous appendage, although there are continuous variations in these taxa. These variabilities pose serious challenges to species delimitation. Therefore, the diversity of seed coat ornamentation can be helpful in species identification and classification. Digitiform subtype is distributed in *I. chekiangensis*, *I. huangyanensis*, *I. chloroxantha*, and *I. platysepala*, and the surface of the digitiform protrusions is distinct from the sect. *Racemosae*, showing dense granular appendages, while the carinate subtype is found in *I. noli-tangere*, *I. nasuta*, and *I. longialata*.

Seed coat ornamentation in *I.* sect. *Uniflorae* includes fine reticulate subtype, areolate subtype, squamalate subtype, cristate subtype, threaded subtype, and granulate subtype. The granulate subtype is only seen in *I. balsamina* and *I. muscicola*. The threaded subtype occurred in *I. tripetala*, *I. burtonii*, and *I. gardnerianais*. The squamalate subtype is a unique seed coat ornamentation of *I.* sect. *Uniflorae* with high taxonomic significance. The squamalate subtype existed in *I. mengtszeana*, *I. monticola*, *I. napoensis*, etc., which are endemic to China with high morphological similarity and clustered in a branch in the phylogenetic tree. The seed coat ornamentation may provide an important reference for future taxonomic treatment and species identification of this taxon. The cristate subtype was distributed in *I. walleriana*, *I. aureliana*, *I. psedoviola*, *I. patula*, and *I. xanthina*, where the protrusions in the former three species are crest like, and the protrusions of *I. xanthina* are spirally thickened.

In conclusion, some seed characteristics were found to be useful for systematic evaluations, such as primary ornamentation, seed shape, and secondary ornamentation, since they showed a high diversity and were useful for distinguishing different clades, helping separate the taxa within them. As the most common ornamentation of the *Impatiens*, the reticulate type is scattered in *Impatiens* and has certain significance for taxonomy. For example, fine reticulate subtype, ellipsoid seed, and clavate capsule are common features of the *I.* subgen. *Clavicarpa*. Digitiform ornamentation occurs in sect. *Racemosae* and sect. *Impatiens*, but the surface morphology of the digitiform ornamentation shows greater variability and can be used for the taxonomic identification of these two groups. Threaded ornamentation appears in sect. *Semeiocardium* and sect. *Uniflorae*, the threaded ornamentation in the former is tubular but filamentous in the latter. This ornamentation can be used to distinguish the two groups. Squamalate ornamentation is unique to some taxa of the sect. *Uniflorae* and can be used as a basis for their classification.

Compared with other organs except flowers, in addition to results of previous studies, the seeds are more conserved with greater potential for classification. The seed morphology of *Impatiens* is taxonomically informative at both the section and the species levels, which has important implications for the taxonomy. Similarly, its rich and variable micromorphological diversity can form an important basis for exploring the phylogenetic relationships among taxa within *Impatiens*. Moreover, an identification key to the subgenera and sections of *Impatiens* is proposed.

1a One seed per locule; seed ellipsoid; fine reticulate subtype; no protrusions on the surface of seed coat……*I.* subgen. *Clavicarpa*

1b Numerous seeds per locule (rarely one); seed oblong, ellipsoid, subspheroid or ovate; areolate subtype, striate subtype, digitiform subtype, clustered subtype, carinate subtype, squamalate subtype, cristate subtype, threaded subtype, granulate subtype (rarely fine reticulate subtype), protrusions on the surface of seed coat…….. 2 *I.* subgen. *Impatiens*

2a One seed per locule; capsule obconic (rarely cylindrical)……… *I.* sect. *Semeiocardium*

2b Many seeds per locule; capsule linear, fusiform or clavate……. 3

3a Capsule clavate, linear, cylindrical……. 4

3b Capsule fusiform…….. 6

4a Capsule clavate……sect. *Tuberosae*

4b Capsule linear, cylindrical……. 5

5a Seed ellipsoid or ovate; areolate subtype, striate subtype, clustered subtype or digitiform subtype, sparsely granular appendages or holes on digitiform protrusions…….. sect. *Racemosae*

5b Seed ellipsoid (rarely ovate); fine reticulate subtype, striate subtype, carinate subtype or digitiform subtype, numerous cuticular granules on digitiform protrusions………. sect. *Impatiens*

6a Seed ellipsoid or ovate; appendicular type (rarely reticulate type), squamalate, cristate, threaded, granulate ornamentation on the surface of seed coat with few exceptions…….. sect. *Uniflorae*

6b Seed subspheroid; reticulate type, densely granulate or striate ornamentation on the surface of seed coat……… 7

7a Seed medium or larger in size; fine reticulate subtype or striate subtype………. sect. *Scorpioidae*

7b Seed medium in size; striate subtype………. sect. *Fasciculatae.*

### Evolution of Seedcoat Ornamentation

In this study, we traced the evolution of five seed characters and detected the evolutionary trends of seed coat ornamentation with a well-resolved phylogenetic framework. Of the various seed coat types, the reticulate type is the most common, since it was observed in 60 species. Ancestral character state reconstructions suggested that the reticulate type was the ancestral state in *Impatiens*, which is congruent with previous reports (Song et al., 2005). The fine reticulate subtype first occurred in *I.* subgen. *Clavicarpa* (Figure 9, clade I), and was located at the relative basal positions on the molecular phylogenetic tree. In this subgenus, the fine reticulate subtype is convergent and conserved, serving as a synapomorphy. Janssens et al. (2009) indicate that the center of the origin for *Impatiens* is Southwest China, and the distribution center of subgen. *Clavicarpa* in southwestern China and northern Vietnam, is in the low-elevation areas. Consequently, we speculate that the distribution center of the fine reticulate subtype is southwest China.

Various more complex protrusive subtypes appeared in the middle and the apex of the molecular phylogenetic tree. In *I.* sect. *Semeiocardium* (Figure 9, clade II), the phylogenetic tree was divided into two branches. The branch at the base (Figure 9, clade IIA) retained the same ornamentation as subgen. *Clavicarpa*, and in the other branch (Figure 9, clade IIB), the primary ornamentation evolved into the appendicular type, including threaded subtype and granulated subtype. Subsequently, this type is lost in sect. *Racemosae*, sect. *Impatiens*, sect. *Scorpioidae*, and sect. *Tuberosae*, and appear as other types of appendages at the top of the phylogenetic tree. At the same time, the striate subtype is a derived state in *I. namkatensis*. Most species of sect. *Semeiocardium* are found in limestone areas, in Subclade B we found that the floral characters show highly homoplasy, lateral united petals connate into 2-lobed lamella, while seed coat ornamentation showed great variation (Figure 9, cladeII). It indicates that different evolutionary mechanisms between flowers and seeds might have happened in the evolution.

Protrusive type appeared in the middle and apex of the evolutionary tree. In *I.* sect. *Racemosae* (Figure 9, clade III), the periclinal cell walls of epidermal cells of the seed coat in some species are convex, forming a negative network, and the periclinal cell walls of some epidermal cells of the seed coat in some species are elevated significantly higher, forming finger-like protrusions. A small number of species in this group retained fine reticulate subtypes such as *I. blinii*. In sect. *Impatiens* (Figure 9, clade VI), while species from the basal branch (Figure 9, cladeVIA) all have digitiform subtypes, and are concentrated on a small branch of sect. *Impatiens* in the molecular phylogenetic tree. The striate subtype is a synapomorphy for the other branches (Figure 9, clade VIB). In this branch, the *I. davidii*, *I. longshanensis*, and *I. dicentra* reversed to the fine reticulate subtype, and the *I. noli-tangere*, *I. nasuta*, *I. longialata*, and *I. capensis* evolved into carinate subtype. In sect. *fasciculatae* (Figure 8, clade IV), sect. *Tuberosae* (Figure 9, clade V), and sect. *Scorpioidea* (Figure 9, clade VII), the primary ornamentations are all reticulate type, retaining the original state of seed coat ornamentation.

In *I.* sect. *Uniflorae* (Figure 9, clade VIII), in addition to the fine reticulate subtype and areolate subtype ornamentation, some subtypes, such as squamalate subtype, cristate subtype, and threaded subtype, have evolved. The squamalate subtype is concentrated in a small branch of sect. *Uniflorae* in the evolutionary tree (Figure 9, cladeVIIIA), and the other two subtypes are scattered in sect. *Uniflorae*. Interestingly, species with squamalate seed coat ornamentation are all distributed in China, which means that the seed coat ornamentation of sect. *Uniflorae* in China is highly conserved, but the seed coat ornamentation of sect. *Uniflorae* in Africa has undergone dramatic changes.

Higher diversity of seed ornamentation was recorded for the *I.* sect. *Uniflorae* while the type of fruit is generally fusiform. This shows that diversity in one character does not imply diversity in another, and that one character and its states may vary tremendously, as other characters remain stable (Liu et al., 2016). This indicates that diversification of seed ornamentation in *Impatiens* has not been stepwise or predictable, but random and abrupt. Mapping the seed characters to the phylogenetic tree clearly showed that seed coat ornamentation of *Impatiens* experienced a complex evolution, evolving from a reticulate type into more complex types with frequent changes within the genus.

### Insights of Ecological Adaptation of Seed Coat Ornamentation

As is well-known, the gynoecium expands gradually after the flower opens, forming different shapes of the mature capsule. The capsule dehisces with sudden explosions, leading to dispersal of seeds, thus making *Impatiens* species unique from other plants (Gogoi et al., 2018). According to this mode of seed dispersal, we can thus speculate that the diversity of seed coat ornamentation is associated with the mechanism of the seed dispersal as well as its habitat. On the one hand, complex seed coat ornamentation may be an adaptation to the environment and transmission mechanisms. For example, the *I.* subgen. *Clavicarpa* is perennial, and is usually found in understories, in shaded moist places, and thus does not require complex seed coat ornamentation to adapt to its environment. Hence, its seed coat ornamentation is the simple fine reticulate subtype. However, *I.* sect. *Semeiocardium* is mostly distributed in dry areas; hence, the surface of the seeds is sparsely covered with hollow tubular-shaped threaded appendages, which are possibly used to absorb water and keep moisture during seed germination. Furthermore, the scaly seed coat ornamentation has small holes in sect. *Uniflorae*, which may increase the propagation distance of seeds (resembling the fluid dynamics of a golf ball) (Fischer et al., 2021), and increase soil adhesion. In addition, this group usually grows along riversides or by waters in shaded places; hence, this ornamentation may enhance seed floating and its ability to achieve long-distance dissemination. On the other hand, the special seed coat ornamentation may help seeds to attach to the outside of an animal for a period and complete seed dispersal. For instance, cristate ornamentation in sect. *Uniflorae* may have a function in adhering the seeds to potential vectors like birds for long-distance dispersal, because the cristate appendages are appressed to the testa when dry but spread when wet (Fischer et al., 2021). Long filamentous thread in *I. tripetala* (sect. *Uniflorae*) may help seeds adhere to the surface of ungulates fur, with a similar function to the cristate ornamentation. However, the function of seed coat still needs more experimental evidence and field investigation to enhance the understanding of its role in seed dispersal within this genus. In addition, based on years of field observation, we found that some insects such as ants may also be involved in the seed dispersal of this genus, but the specific mechanism of dispersal and the role of insects in dispersal need further investigation and research. Chen (2016) showed that seedcoat ornamentation can help maintain seed viability, inhibit seed germination, and promote seedling growth. The relationship between seed coat ornamentation and seed germination in *Impatiens* deserves further investigation to understand the function of seed coat ornamentation.

## Conclusion

We comprehensively studied the seed micromorphology of *Impatiens* based on extensive taxon sampling and found that the genus holds an extremely high diversity of seed coat ornamentation; it is taxonomically informative at both the subgenus and the species levels, which has important implications for the taxonomy. The seed characters, such as seed shape, seed coat ornamentation, are important not only for taxonomic but also for evolutionary and ecological studies. The seed coat can be divided into reticulate type, protrusive type, and appendicular type, and they can be further subdivided into 10 subtypes: fine reticulate subtype, areolate subtype, striate subtype, digitiform subtype, clustered subtype, carinate subtype, squamalate subtype, cristate subtype, threaded subtype, and granulate subtype. We reconstructed the ancestral states of five seed characters of the *Impatiens* and found that the seed characters of shape, primary ornamentation, and anticlinal cell wall could be identified as unambiguous, and that seed coat ornamentation of *Impatiens* may have undergone complex evolutionary mechanisms. In addition, we speculated that the extremely high diversity of seed coat ornamentation in this genus is possibly related to the seed dispersal and environmental adaptation. The variable seed coat ornamentation may be the adaptation of plants to the environment and transmission and may help seeds to attach to the outside of an animal for seed dispersal.

## Data Availability Statement

The datasets presented in this study can be found in online repositories. The names of the repository/repositories and accession number(s) can be found in the article/Supplementary Material.

## Author Contributions

Y-YC and G-WH contributed to the conceptualization. SP and HJ contributed to the methodology. SP and Y-XS contributed to the software and formal analysis. SP and JR contributed to the investigation. Y-XS contributed to the writing—original draft preparation. Y-YC, G-WH, and FM contributed to the writing—review and editing. All authors have read and agreed to the published version of the manuscript.

## Conflict of Interest

The authors declare that the research was conducted in the absence of any commercial or financial relationships that could be construed as a potential conflict of interest.

## Publisher’s Note

All claims expressed in this article are solely those of the authors and do not necessarily represent those of their affiliated organizations, or those of the publisher, the editors and the reviewers. Any product that may be evaluated in this article, or claim that may be made by its manufacturer, is not guaranteed or endorsed by the publisher.
