# Supplementary material for: Evolution and Taxonomic Significance of Seed Micromorphology in Impatiens (Balsaminaceae)
Source: Front Plant Sci. 2022 Feb 16;13:835943. doi: 10.3389/fpls.2022.835943 (PMC8889038; doi:10.3389/fpls.2022.835943)
Supplement: Supplementary file 1 [file Data_Sheet_1.docx]

Supplementary Material

**Evolution and taxonomic significance of seed micromorphology in *Impatiens* (Balsaminaceae)**

**Yong-Xiu Song^1,2,†^, Shuai Peng^2,3,4,†^, Fredrick Munyao Mutie^2,3,4^, Hui Jiang^2,3,4^, Jing Ren^1,2^,Yi-Yan Cong^1,*^, Guang-Wan Hu^2,3,4,*^**

^1^College of Life Sciences, Hunan Normal University, Changsha, China

^2^Core Botanical Gardens/Wuhan Botanical Garden, Chinese Academy of Sciences, Wuhan, China

^3^Sino-Africa Joint Research Center, Chinese Academy of Sciences, Wuhan, China

^4^University of Chinese Academy of Sciences, Beijing, China

*Correspondence:
Yi-Yan Cong
[congyiyan2004@aliyun.com](mailto:congyiyan2004@aliyun.com)

Guang-Wan Hu
[guangwanhu@wbgcas.cn](mailto:guangwanhu@wbgcas.cn)

^†^These authors contributed equally to this work

# Supplementary Data

## Supplementary Figures


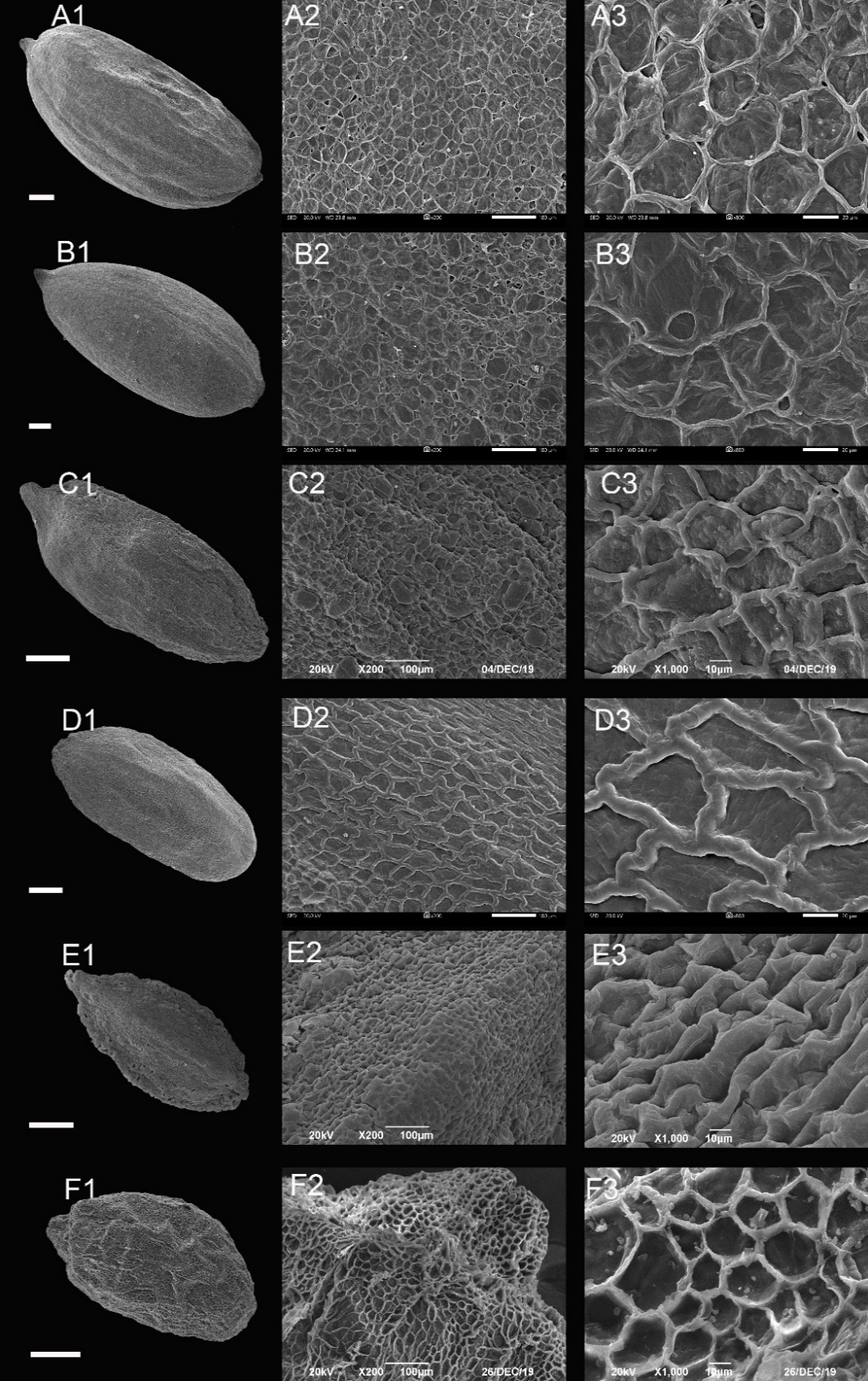


**Supplementary Figure 1**. **Scanning electron microscope images of seeds in *Impatiens*. (A1-F1)** Whole view; **(B2-F2, C3-F3)** Partial view. **(A1-A3)** *Impatiens purpureifolia*, **(B1-B3)** *Impatiens clavigera*, **(C1-C3)** *Impatiens guizhouensis*, **(D1-D3)** *Impatiens pritzelii*, **(E1-E3)** *Impatiens omeiana*, **(F1-F3)** *Impatiens stenosepala.* Scale bars:**(A1-F1)** = 500 μm.


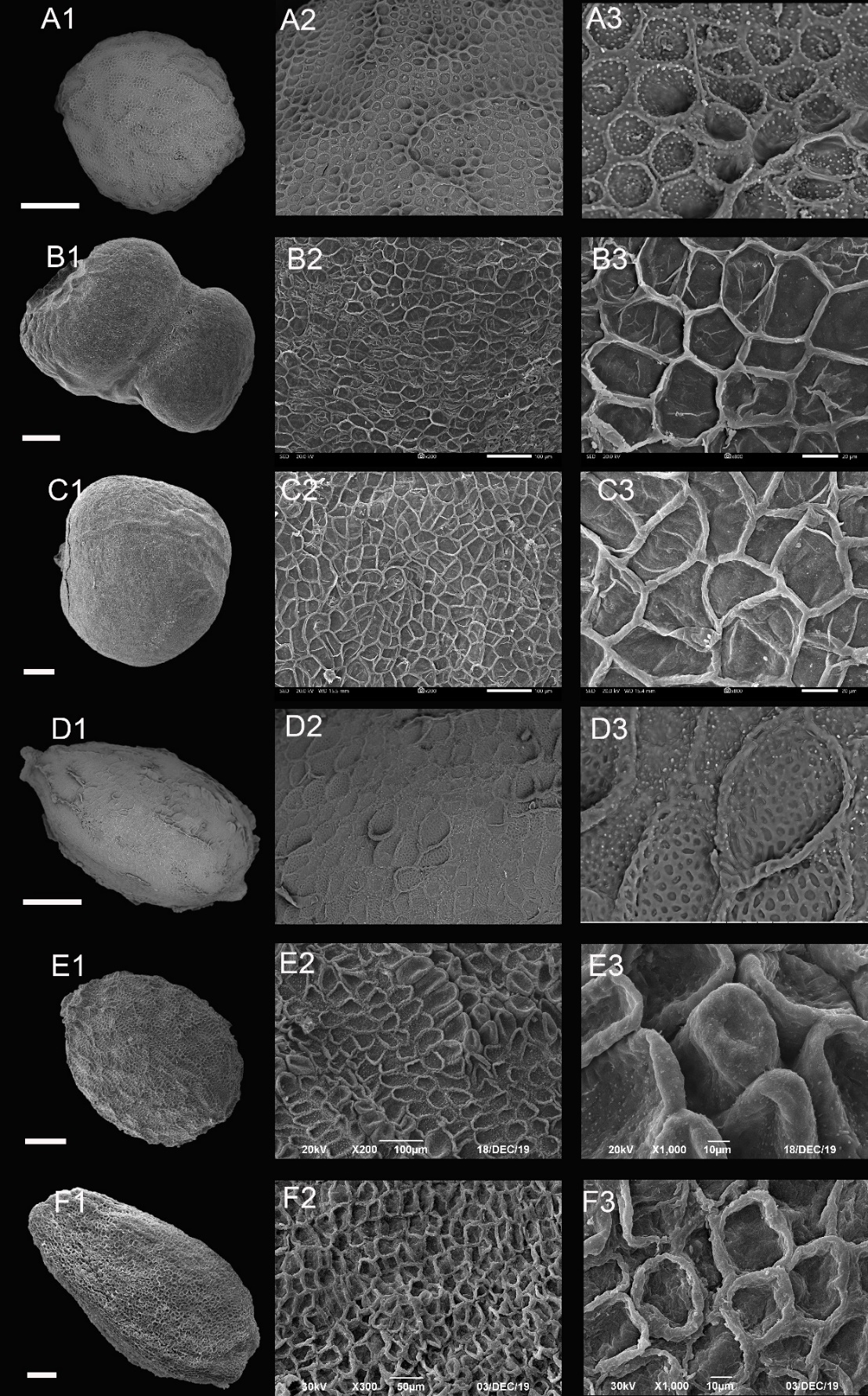


**Supplementary Figure 2.** **Scanning electron microscope images of seeds in *Impatiens*. (A1-F1)** Whole view; **(B2-F2, C3-F3)** Partial view. **(A1-A3)** *Impatiens xishuangbannaensis*, **(B1-B3)** *Impatiens wenshanensis*, (C1-C3) *Impatiens linearisepala*, (D1-D3) *Impatiens stenantha*, **(E1-E3)** *Impatiens blinii*, **(F1-F3)** *Impatiens davidii*. Scale bars: **(A1-E1)** = 500 μm, **(F1)** = 200 μm.


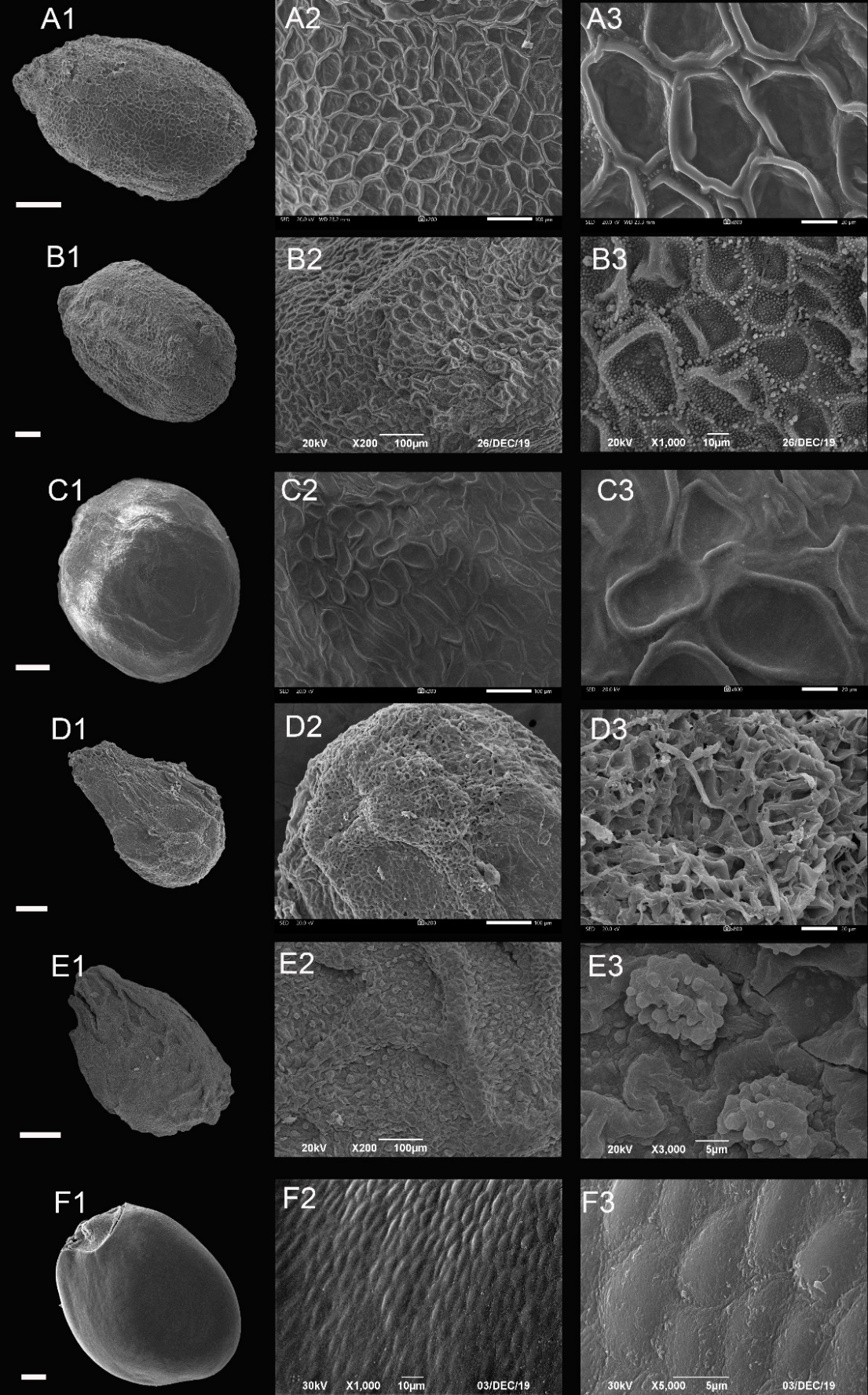


**Supplementary Figure 3.** **Scanning electron microscope images of seeds in *Impatiens*.** **(A1-F1)** Whole view; **(B2-F2, C3-F3)** Partial view. **(A1-A3)** *Impatiens longshanensis*, **(B1-B3)** *Impatiens dicentra*, **(C1-C3)** *Impatiens rubrostriata*, **(D1-D3)** *Impatiens stuhlmannii*, **(E1-E3)** *Impatiens aquatilis*, **(F1-F3)** *Impatiens chinensis*. Scale bars: **(A1, C1)** = 500 μm, **(B1, D1, F1)** = 200 μm.


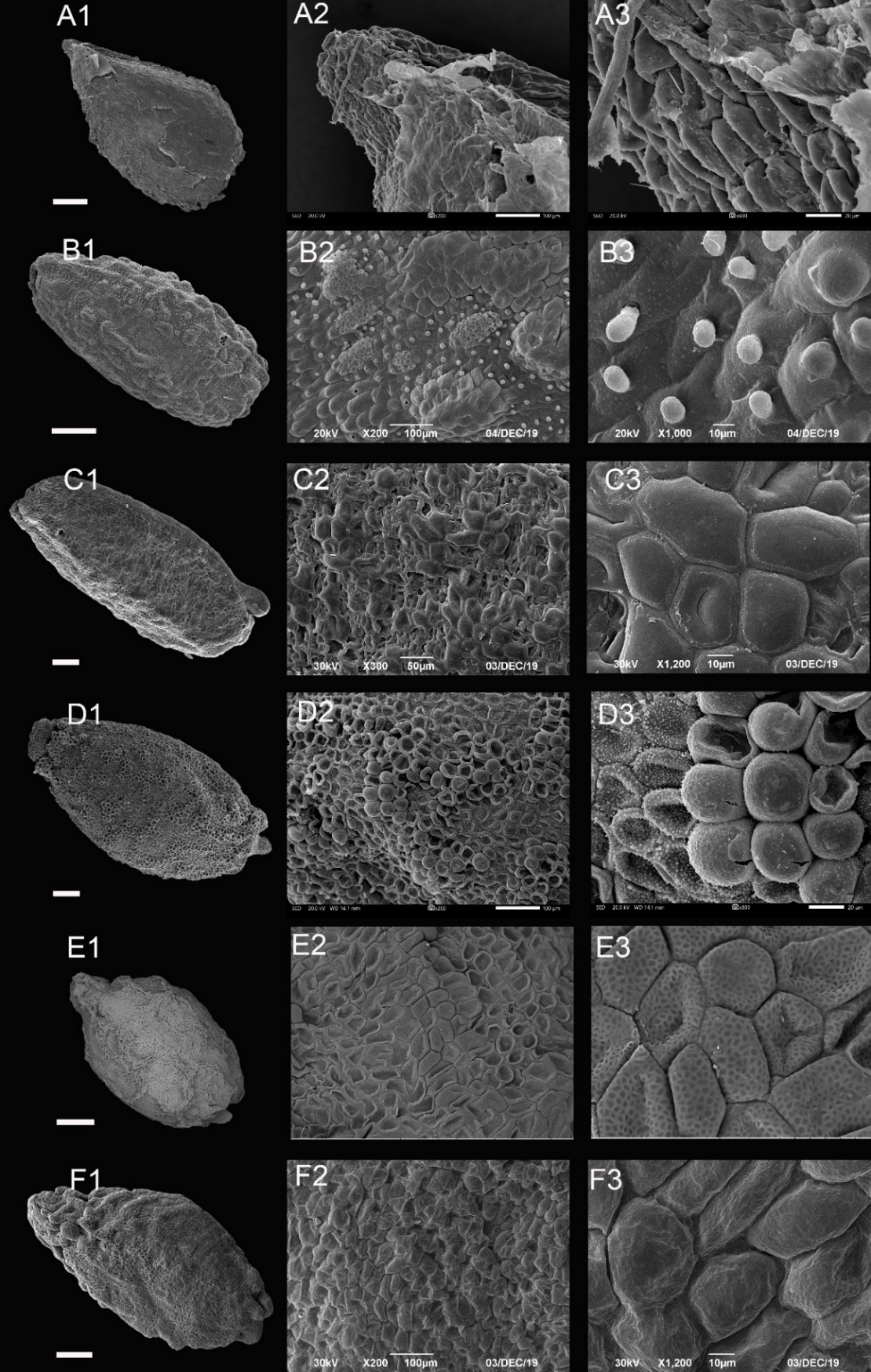


**Supplementary Figure 4.** **Scanning electron microscope images of seeds in *Impatiens*. (A1-F1)** Whole view; **(B2-F2, C3-F3)** Partial view. **(A1-A3)** *Impatiens meruensis*, **(B1-B3)** *Impatiens fragicolor*, **(C1-C3)** *Impatiens siculifer*, **(D1-D3)** *Impatiens chimiliensis*, **(E1-E3)** *Impatiens uliginosa*, **(F1-F3)** *Impatiens jinggangensis*. Scale bars: **(A1, B1, E1, F1)** = 500 μm, **(C1, D1)** = 200 μm.


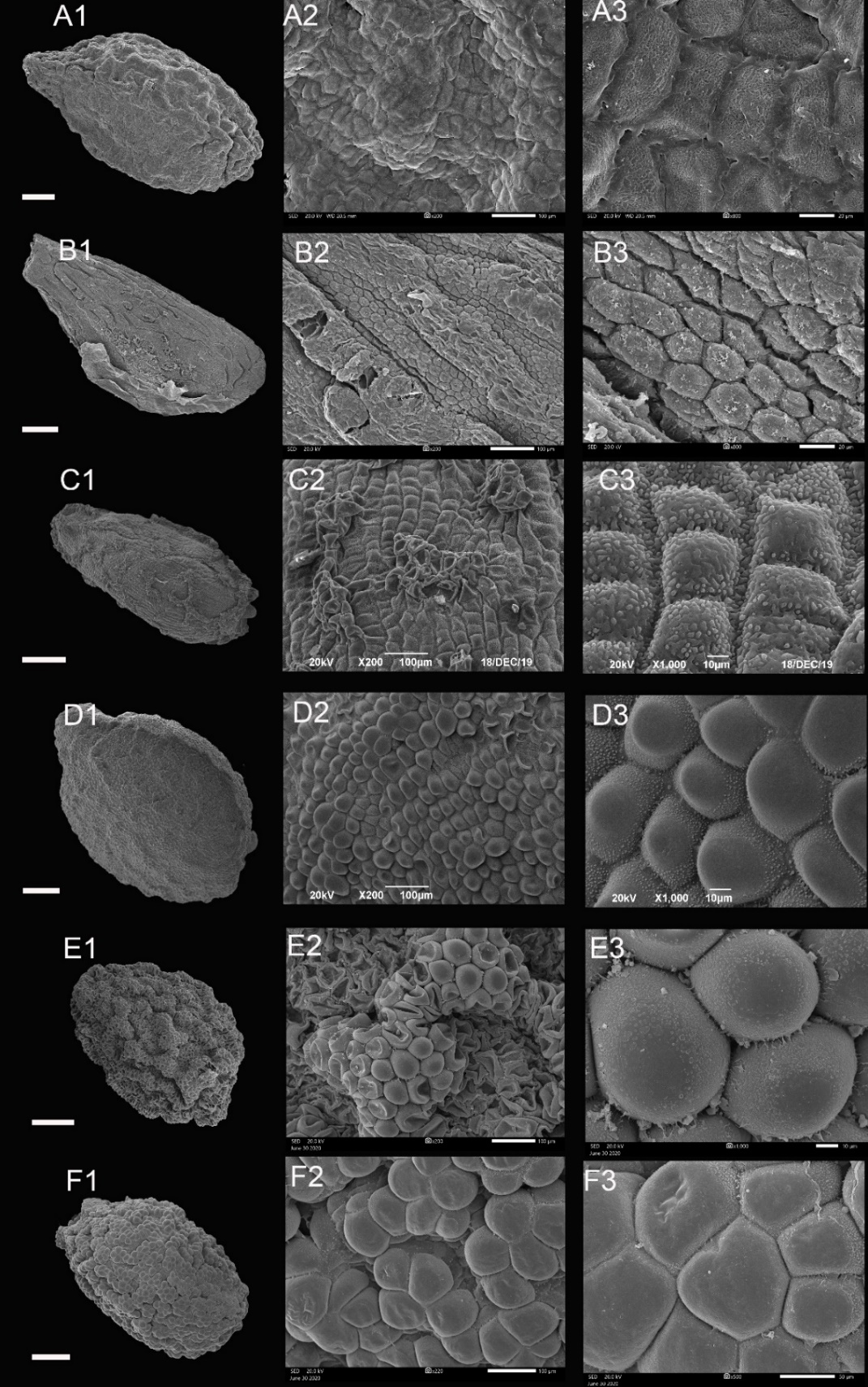


**Supplementary Figure 5.** **Scanning electron microscope images of seeds in *Impatiens*. (A1-F1)** Whole view; **(B2-F2, C3-F3)** Partial view. **(A1-A3)** *Impatiens tsangshanensis*, **(B1-B3)** *Impatiens teitensis subsp teitensis*, **(C1-C3)** *Impatiens poculifer*, **(D1-D3)** *Impatiens soulieana*, **(E1-E3)** *Impatiens reptans*, **(F1-F3)** *Impatiens piufanensis*. Scale bars:**(A1-F1)** = 500 μm.


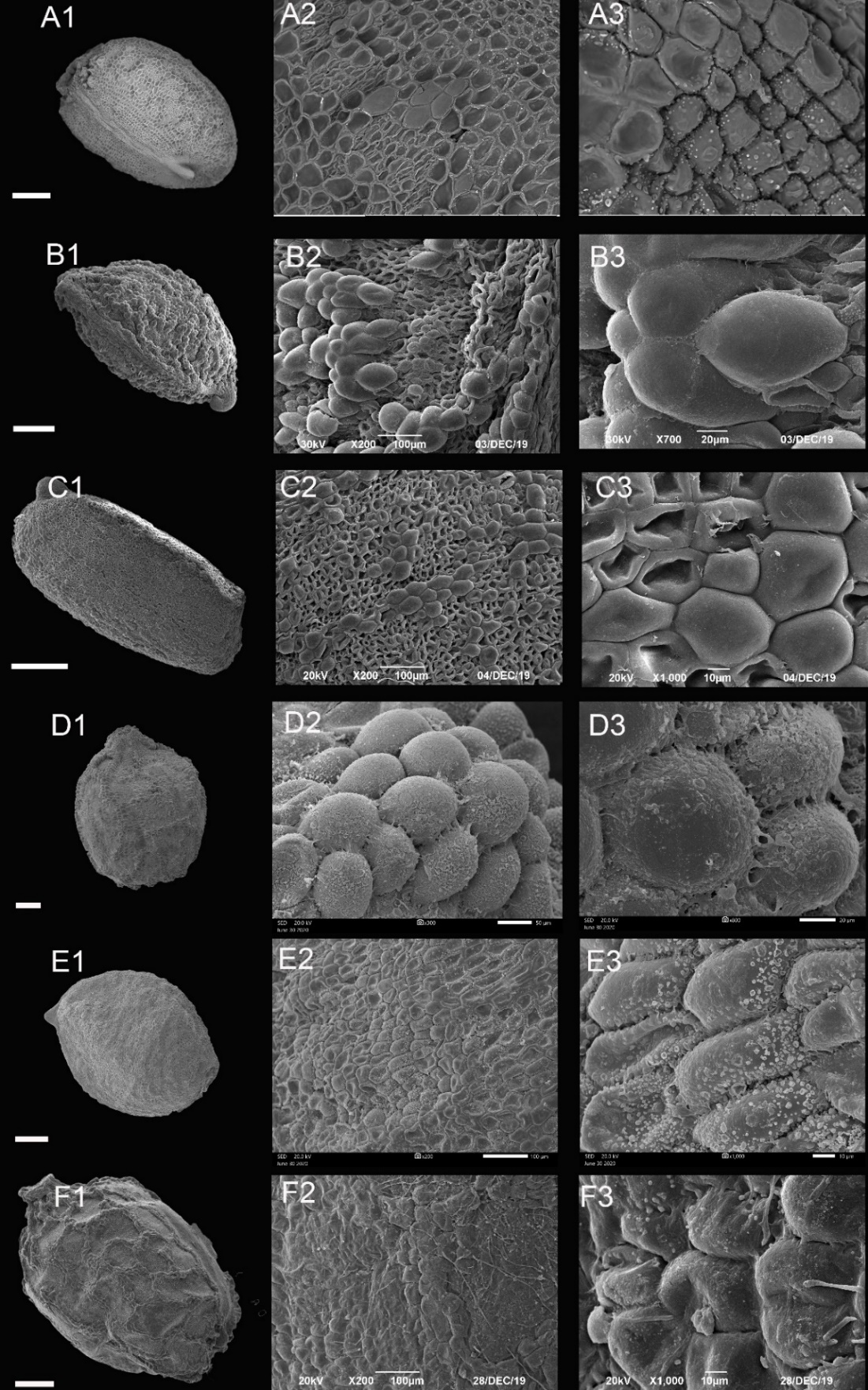


**Supplementary Figure 6.** **Scanning electron microscope images of seeds in *Impatiens*. (A1-F1)** Whole view; **(B2-F2, C3-F3)** Partial view. **(A1-A3)** *Impatiens lepida*, **(B1-B3)** *Impatiens commelinoides*, **(C1-C3)** *Impatiens meyana*, **(D1-D3)** *Impatiens bodinieri*, **(E1-E3)** *Impatiens macrovexilla*, **(F1-F3)** *Impatiens blepharosepala*. Scale bars:**(A1-F1)** = 500 μm.


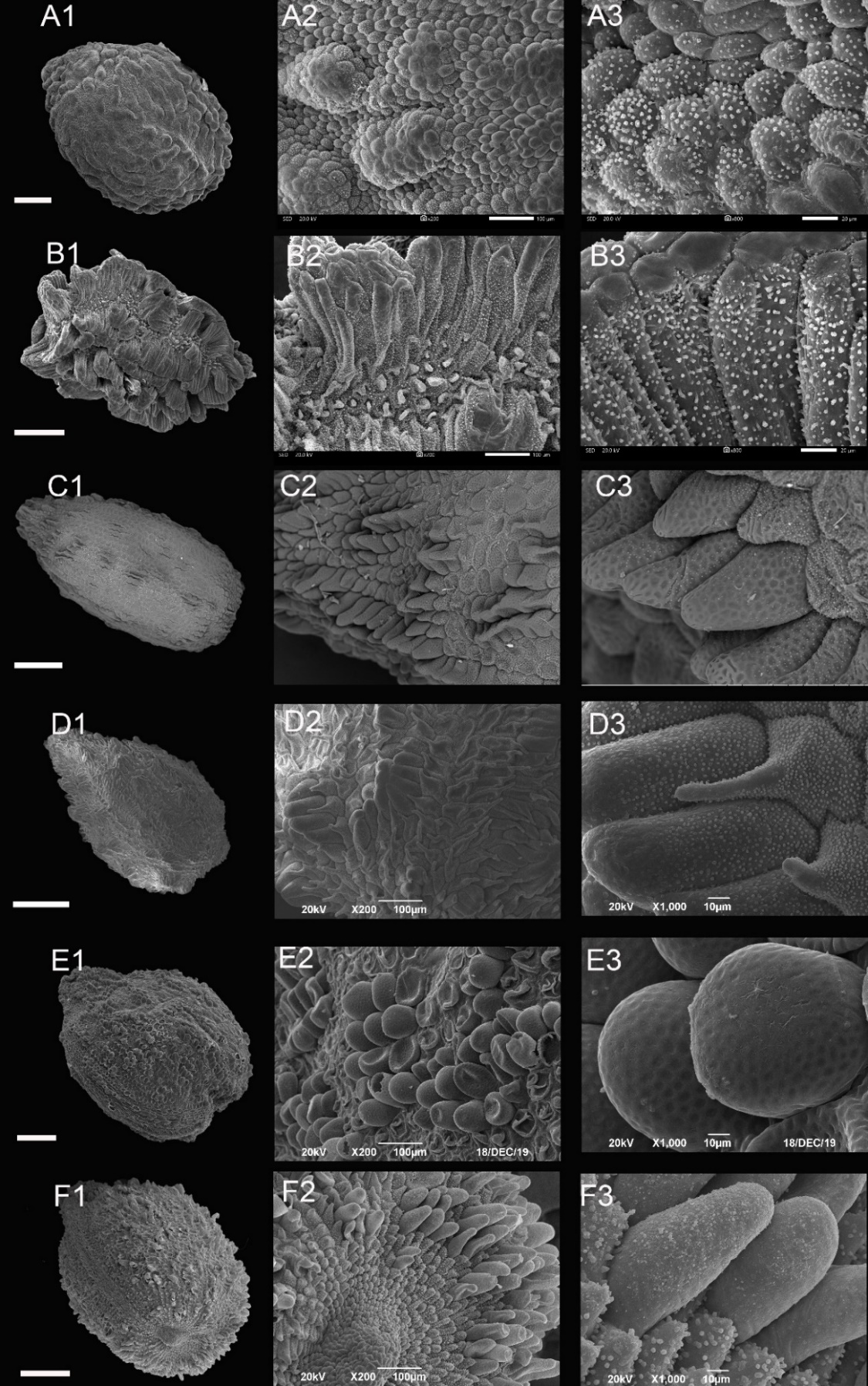


**Supplementary Figure 7.** **Scanning electron microscope images of seeds in *Impatiens*. (A1-F1)** Whole view; **(B2-F2, C3-F3)** Partial view. **(A1-A3)** *Impatiens hunanensis*, **(B1-B3)** *Impatiens falcifer*, **(C1-C3)** *Impatiens radiata*, **(D1-D3)** *Impatiens racemosa*, **(E1-E3)** *Impatiens siculifer* var *mitis*, **(F1-F3)** *Impatiens nyimana*. Scale bars:**(A1-F1)** = 500 μm.


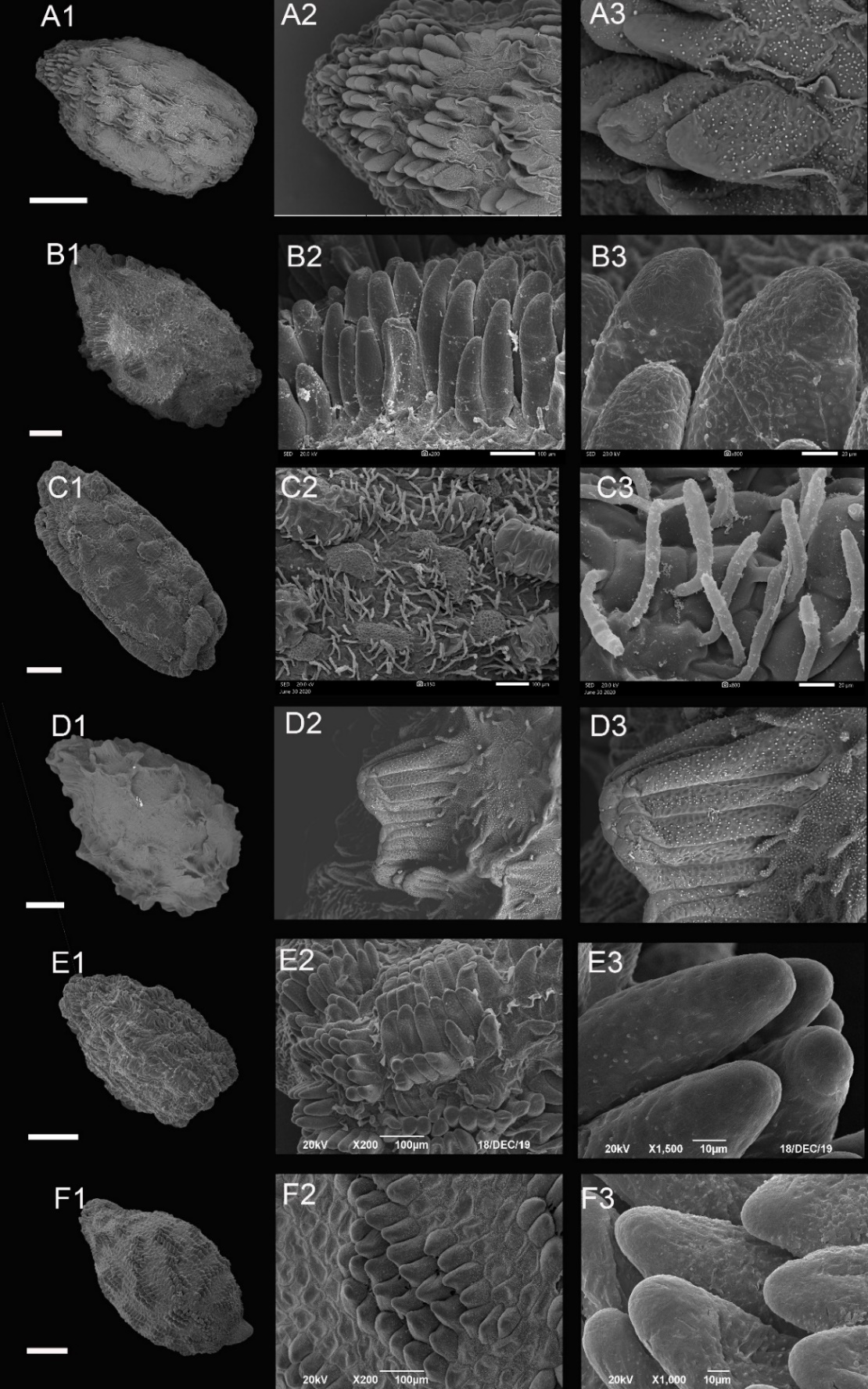


**Supplementary Figure 8.** **Scanning electron microscope images of seeds in *Impatiens*. (A1-F1)** Whole view; **(B2-F2, C3-F3)** Partial view. **(A1-A3)** *Impatiens holocentra*, **(B1-B3)** *Impatiens cyathiflora*, **(C1-C3)** *Impatiens sterilis*, **(D1-D3)** *Impatiens pseudokingii*, **(E1-E3)** *Impatiens rectangula*, **(F1-F3)** *Impatiens bahanensis*. Scale bars:**(A1-F1)** = 500 μm.


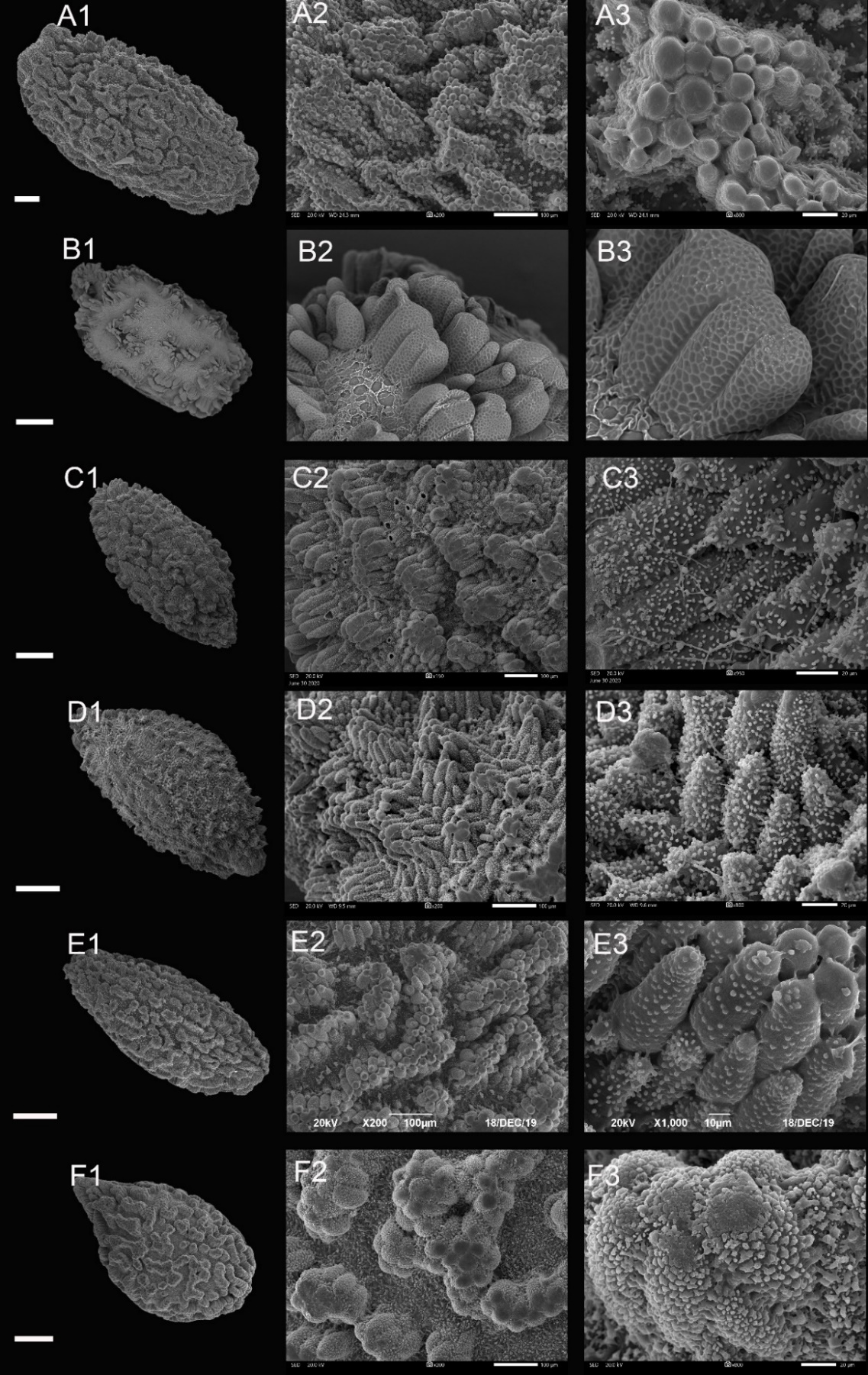


**Supplementary Figure 9.** **Scanning electron microscope images of seeds in *Impatiens*. (A1-F1)** Whole view; **(B2-F2, C3-F3)** Partial view. **(A1-A3)** *Impatiens fenghwaiana*, **(B1-B3)** *Impatiens pianmaensis*, **(C1-C3)** *Impatiens platysepala*, **(D1-D3)** *Impatiens chloroxantha*, **(E1-E3)** *Impatiens chekiangensis*, **(F1-F3)** *Impatiens delavayi*. Scale bars: **(B1-F1)** = 500 μm, **(A1)** = 200 μm.


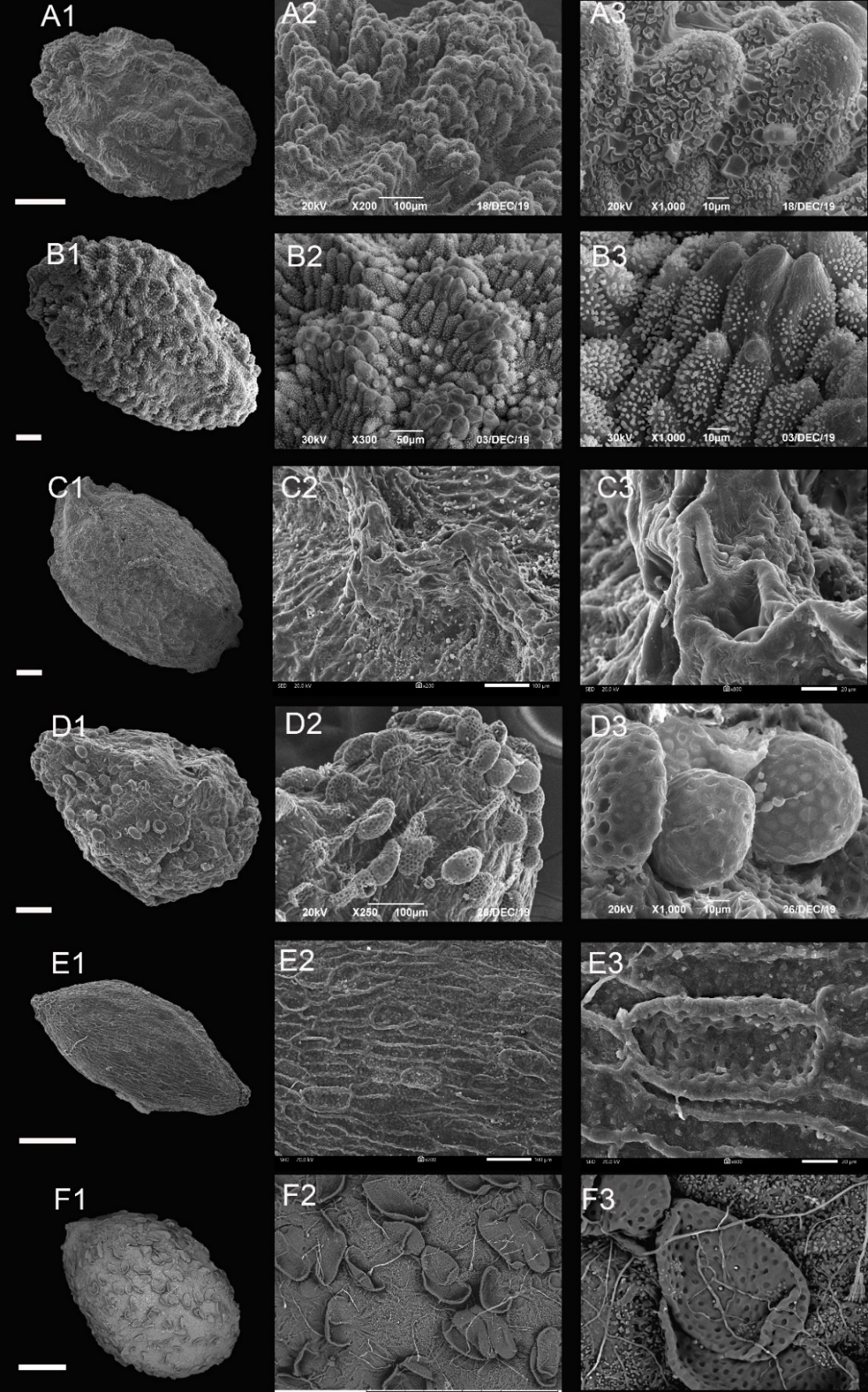


**Supplementary Figure 10.** **Scanning electron microscope images of seeds in *Impatiens*. (A1-F1)** Whole view; **(B2-F2, C3-F3)** Partial view. **(A1-A3)** *Impatiens apsotis*, **(B1-B3)** *Impatiens wuyuanensis*, **(C1-C3)** *Impatiens nasuta*, **(D1-D3)** *Impatiens yingjiangensis*, **(E1-E3)** *Impatiens mengtszeana*, **(F1-F3)** *Impatiens tomentella*. Scale bars: **(A1, C1, E1, F1)** = 500 μm, **(B1, D1)** = 200μm.


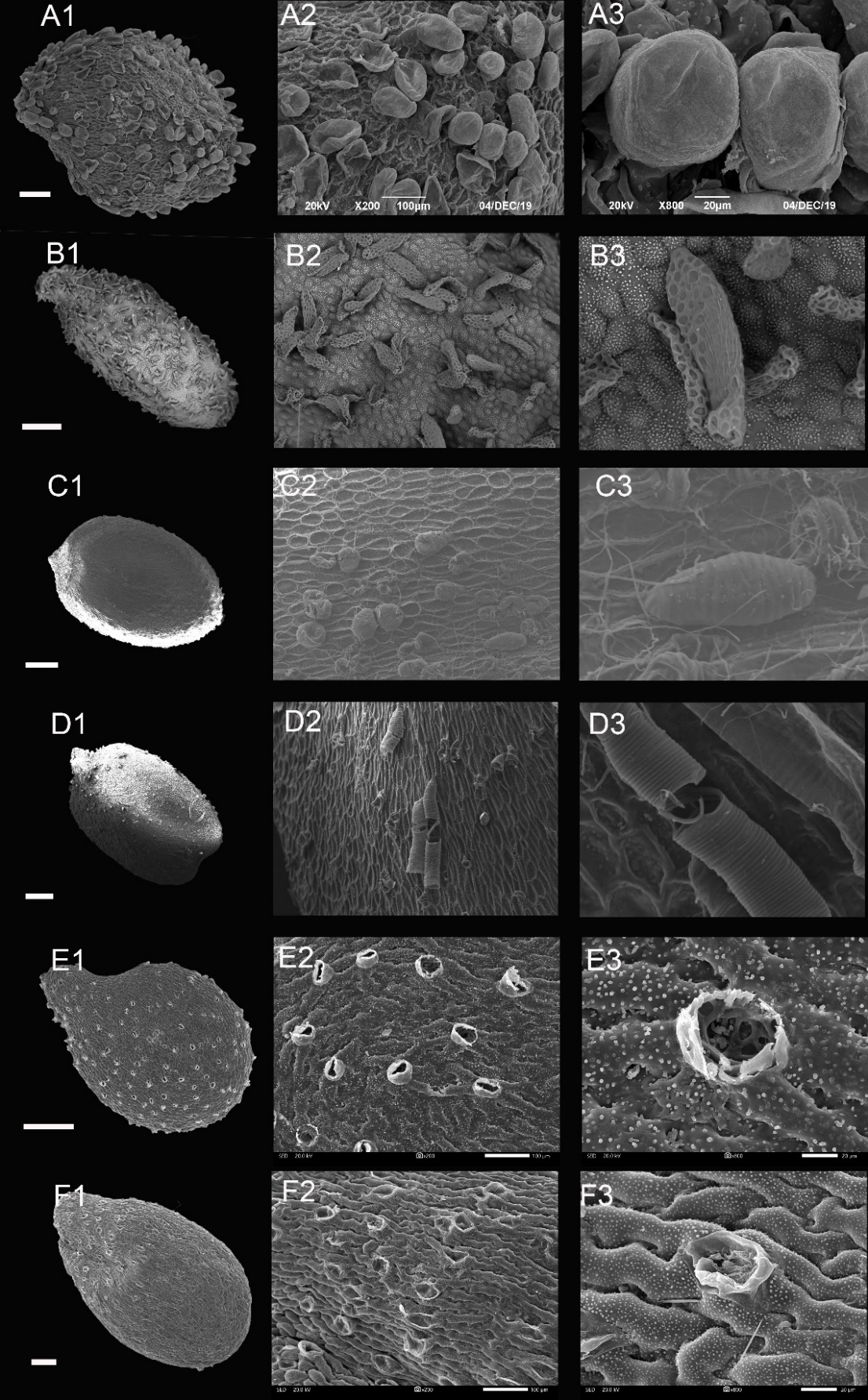


**Supplementary Figure 11.** **Scanning electron microscope images of seeds in *Impatiens*. (A1-A3)** *Impatiens napoensis*, **(B1-B3)** *Impatiens kerriae*, **(C1-C3)** *Impatiens pingxiangensis*, **(D1-D3)** *Impatiens morsei*, **(E1-E3)** *Impatiens walleriana*, **(F1-F3)** *Impatiens aureliana*. Scale bars: **(B1-E1)** = 500 μm, **(A1, F1)** = 200μm.

**Supplementary Table 4. Reference**

Cai, X. Z., Yi, R. Y., Zhuang Y. H., Cong, Y. Y., Kuang, R. P., and Liu, K. M. (2013). Seed coat micromorphology characteristics of *Impatiens* L. and its systematic significance. *Acta Hortic. Sin*. 40, 1337-1348. [doi](https://doi): 10.16420/j.issn.0513-353x.2013.07.014

Fischer, E., Rahelivololona, M. E., and Abrahamczyk, S. (2017). *Impatiens galactica* (Balsaminaceae), a new spurless species of section Trimorphopetalum from Madagascar. *Phytotaxa*- 298, 269-276. doi: 10.11646/phytotaxa.298.3.6

Lu, Y. Q. and Chen, Y. L. (1991). Seed morphology of *Impatiens* L. (Balsaminaceae) and its taxonomic significance. *Acta Phytotax. Sin.* 29, 252-257.

Najberek, K., Olejniczak, P., Berent, K., Gąsienica-Staszeczek, M., and Solarz, W. (2020). The ability of seeds to float with water currents contributes to the invasion success of *Impatiens balfourii* and *I. glandulifera*. *J. Plant Res.* 133, 649664. [doi: 10.1007/s10265-020-01212-0](https://doi.org/10.1007/s10265-020-01212-0)

Rewicz, A., Adamowski, W., Borah, S., and Gogoi, R. (2020a). New data on seed coat micromorphology of several *Impatiens* spp. from Northeast India. *Acta Soc. Bot. Pol.* 89, Article-89312. [doi: 10.5586/asbp.89312](https://doi.org/10.5586/asbp.89312)

Rewicz, A., Myśliwy, M., Adamowski, W., Podlasiński, M., and Bomanowska, A. (2020b). Seed morphology and sculpture of invasive *Impatiens capensis* Meerb. from different habitats. *PeerJ* 8, e10156. [doi.org/10.7717/peerj.10156](https://doi.org/10.7717/peerj.10156)

Song, Y., Yuan, Y. M., and Küpfer, P. (2005). Seedcoat micromorphology of *Impatiens* (Balsaminaceae) from China. *Bot. J. Linn. Soc.* 149, 195-208. doi:10.1111/j.1095-8339.2005.00436.x.

Utami, N., and Shimizu, T. (2005). Seed morphology and classification of *Impatiens* (Balsaminaceae). *Blumea-Biodiversity, Evolution and Biogeography of Plants*, 50, 447-456. doi: 10.3767/000651905X622699

Xia, C. Y. (2020). Phylogeny study and taxonomic revision of *Impatiens* subg. *Clavicarpa* [Master thesis]. Chongqing Southwest University, China.
